# Efficacy, effectiveness and safety of medical cannabis in PTSD: a scoping review

**DOI:** 10.1186/s42238-026-00451-7

**Published:** 2026-05-29

**Authors:** Joshua Aviram, Rostislav Belobrov, Shulamit Grinapol, Eyal Fruchter

**Affiliations:** 1 Department of Nursing, Faculty of Health Sciences, Ariel University, Ariel, 40700 Israel; 2 Syqe Medical Ltd, Ha Thiya 14, Tel Aviv, 6816914 Israel; 3 Maale Hacarmel, Mental Health Center, Tirat Carmel, Israel; 4 Psychiatry & Mental Health Division, Rambam Health Care Campus, Haifa, Israel; 5 ICAR collective, Brus Rappaport medical faculty, Technion, Haifa, Israel

**Keywords:** Post-traumatic stress disorder, Cannabinoids, Psychiatry, Medical cannabis, Efficacy, Safety, Scoping review

## Abstract

**Background:**

Although current treatment guidelines recommend against cannabinoids for Post-Traumatic Stress Disorder (PTSD), their use has increased in clinical settings despite fragmented evidence. This review critically examines the efficacy, effectiveness, and safety of cannabinoid-based interventions for PTSD.

**Methods:**

We conducted a scoping review following PRISMA-ScR guidelines. Five databases were searched up to December 22, 2024. Eligible studies included randomized controlled trials (RCTs) and observational studies, investigating cannabinoids in PTSD-diagnosed populations. Screening, extraction, and quality appraisal independently performed. Quality assessed using validated tools. Main outcomes included PTSD symptom severity and adverse events (AEs).

**Results:**

From 1,474 screened titles, 26 studies included: 7 RCTs, 9 prospective, 9 retrospective observational studies, and one unpublished RCT, totaling 3,598 patients. Among the seven RCTs, only one demonstrated a statistically significant reduction in PTSD-related nightmares (nabilone vs. placebo). Two RCTs using inhaled or oral cannabis did not show superiority over placebo. Two additional RCTs evaluating oral THC during fear extinction tasks identified changes in neurobiological activation (e.g., increased ventromedial prefrontal cortex activity or reduced fear renewal) without clinical symptom improvement. The remaining two RCTs involving acute oral CBD administration showed minimal benefit, limited to transient mood or cognitive modulation during trauma recall. Overall, only one of the seven RCTs showed clear clinical efficacy over placebo; the rest showed no significant group differences. Observational studies frequently reported symptom improvements, particularly in nightmares, hyperarousal, sleep, and quality of life; however, these findings are limited by high risk of bias, reliance on self-reported outcomes, and lack of control groups, reducing confidence in causal interpretations. AEs were generally mild (e.g., dry mouth, dizziness). Risk of bias was high in most observational studies and moderate in RCTs.

**Conclusions:**

The current evidence from high-quality RCTs remains insufficient to support clinical use of cannabinoids in treating PTSD. The therapeutic role of cannabinoids in PTSD should be further evaluated through rigorous RCTs.

**Trial registration:**

The review design was developed 'a priori' to data collection initiation but was not registered in PROSPERO prior to initiation.

**Supplementary Information:**

The online version contains supplementary material available at 10.1186/s42238-026-00451-7.

## Introduction

Cannabis consumption has significantly increased in recent years, particularly following its medicalization in many countries across the United States (US) and Europe (Boehnke et al. 2022). In 2020, nearly 3 million patients held a medical cannabis (MC) license, representing a 4.4-fold increase since 2016 (Boehnke et al. 2022). Nonetheless, cannabis and most of its components remain classified as a Schedule I drug, with no proven medical use, high potential for abuse, and lack of accepted safety protocols (DEA 2018). This is due to the absence of the Food and Drug Administration (FDA) approval and the lack of a traditional pharmaceutical development process, despite its growing use in medical settings. This legal status is similar in most territories, regardless of the cannabis legalization or medicalization status, for example, in the European Union (EU), based on the report of the EU Drug Agency (EUDA) (EUDA 2023).

Hence, current treatment guidelines strongly recommend against cannabinoids treatment for Post Traumatic Stress Disorder (PTSD) (Lang et al. 2024), citing the lack of high-quality trials with well-documented safety profiles. These recommendations are informed by earlier and more recent reviews and guidelines, including literature reviews published between 2015 and 2017 (Belendiuk et al. 2015; Kansagara et al. 2017; Steenkamp et al. 2017; Wilkinson et al. 2016), subsequent systematic reviews of cannabinoids for PTSD and psychiatric indications (Rehman et al. 2021; CADTH 2019; Wilson et al. 2026; Stanciu et al. 2021), and recent guideline updates addressing PTSD and related anxiety disorders (Vigod et al. 2025). Overall, these sources consistently emphasize that the clinical evidence remains limited and does not currently support routine cannabinoid use for PTSD.

Cannabis-Based Medicines (CBMs) and whole-plant Medical Cannabis (MC) represent distinct approaches to cannabis-derived treatments. CBMs typically comprise synthetic cannabinoids (e.g., Nabilone and Dronabinol, which are synthetic, or plant derived Δ^9^-Tetrahydrocannabinol [THC]) or purified cannabis botanical derivates (e.g., Nabiximoles, which combines purified THC and purified cannabidiol [CBD], and Epidiolex which is a purified CBD), all of which are administered orally. In contrast, MC is most commonly administrated via inhalation (mainly via vaporizing or smoking), but can also be administered orally as extracts dissolved in vegetable oils (Hazekamp et al. 2013). Notably, whole-plant MC products are frequently considered "full-spectrum", containing over 100 cannabinoids (Berman et al. 2018), alongside other components, such as almost 100 terpenes (Shapira et al. 2019), which increase the affinity of THC to the CB1 receptors (Raz et al. 2023). Through their interaction with the endocannabinoid system, cannabinoids have demonstrated mechanistic potential in influencing PTSD pathophysiology (Neumeister et al. 2013), potentially related to an endocannabinoid deficiency state (Russo 2016), mainly represented by findings of reduced endocannabinoids in the cerebral spinal fluid of migraine patients compared to controls (Sarchielli et al. 2007). Clinically, although based on observational studies, it is assumed that the rationale for MC treatment, is a decrease in PTSD negative symptoms, and especially, nightmares decrease (Hindocha et al. 2020).

The signal for symptomatic improvements that were reported mostly by observational studies (Hindocha et al. 2020) might be explained by the role of cannabinoids in fear extinction mechanism, which is central in the pathophysiology of PTSD (Neumeister et al. 2013). Low doses of THC appear to reduce anxiety and enhance the extinction of fear memories in humans. These effects are mediated by the activation of CB1 receptors within the corticolimbic circuitry, a brain region central to emotional processing (Forsythe and Boileau 2022). Both naturally occurring cannabinoids (such as THC and CBD) and synthetic ones (like Nabilone) seem to bolster the consolidation and retention of extinction training, thereby further reducing conditioned fear responses (Forsythe and Boileau 2022). These potentially be mitigated through exogenous cannabinoid supplementation.

Despite growing clinical use (Mahabir et al. 2020; Sakal et al. 2021) and supportive real-world evidence (RWE) from observational studies (Hindocha et al. 2020), most published literature reviews (Lang et al. 2024; Belendiuk et al. 2015; Kansagara et al. 2017; Steenkamp et al. 2017; Wilkinson et al. 2016; Rehman et al. 2021; CADTH 2019; Cowling and MacDougall 2019; Black et al. 2019; O'Neil et al. 2017; Rodas et al. 2024) and guidelines (Lang et al. 2024) have not endorsed CBMs or MC for PTSD symptom management. This hesitancy is largely attributed to the complexity of the condition and the limited availability of high-quality randomized controlled trials (RCTs), with RWE being almost entirely overlooked. These decisions may also stem from the evidence for the psychoactive impacts of cannabis use, which include feelings of intoxication, stimulation, anxiety, sedation, impaired psychomotor performance, memory deficits, and altered perception, effects that may be particularly undesirable in the context of PTSD treatment. These concerns are typically based on studies of recreational or abusive use rather than structured clinical treatment (Bonn-Miller et al. 2007, 2013; Bremmer et al. 1982; Vandrey et al. 2014; Cougle et al. 2011; Boden et al. 2013; Babson et al. 2013). On the other hand, one review was more positive toward the potential benefits from cannabinoid based treatment, which may play a part in fear extinction and antidepressive effects (Passie et al. 2012).

Regarding the abovementioned cannabis related psychoactive adverse events (AEs), there is evidence that they may be dose dependent. For instance, in a study of the CBM Nabiximols (Sativex) in cannabis-naïve multiple sclerosis patients, higher THC blood levels correlated with increased psychopathological scores, including interpersonal sensitivity, aggressiveness, and paranoia, suggesting that higher doses may elevate psychological risks, especially beyond therapeutic ranges​ (Aragona et al. 2009).

THC is the most abundant phytocannabinoid in many cannabis cultivars and is considered the psychoactive component in the cannabis plant (Atakan 2012), while CBD is considered non-psychoactive and may counteract some THC effects (Colom and Gual 2018). Yet, multiple reports suggest that CBD-rich cannabis may still induce "THC-like" AEs (Hermush et al. 2022; Aran et al. 2021; Aviram et al. 2021; Aviram et al. 2021). with no clear mitigation of psychoactivity observed in 3:1 dose ratio of CBD:THC via vaporization (Englund et al. 2022), and CBD failing to prevent THC-induced driving impairment (Arkell et al. 2019).

Importantly, a clear distinction should be made between non-medical (recreational) and MC use, including differences in intent and usage patterns. Recreational users, mostly younger males, reported significantly higher rates of moderate-to-severe cannabis use disorder (CUD) (7.2%) compared to medical-only users (1.3%) (Lapham et al. 2023). According to World Health Organization (WHO), around 200 million people use cannabis yearly, making it the most common illicit drug, compared to 60 million opioid users globally (WHO 2016). Among United States (U.S.) veterans (2019–2020), 11.9% reported past six-month cannabis use, with only 1.5% holding MC cards, leaving 10% as illicit users (Hill et al. 2021). A 20-year Drug Enforcement Agency (DEA) analysis of confiscated cannabis found THC levels rose from ~ 4% in 1995 to ~ 12% in 2014 (ElSohly et al. 2016) with hashish reaching up to 34% (Mehmedic et al. 2010). similar trends were noted in Europe (Freeman et al. 2018). Hence, evidence from recreational or high-potency cannabis should not be extrapolated to CBMs or MC, which typically involve lower potencies, limited dosing, and medical oversight.

In the U.S., PTSD is the indication for over 5,000 medical cannabis licenses, representing approximately 8.4% of all licensed medical cannabis patients. PTSD patients comprise 6.7% of the UK MC registry (named 'Twenty21') (Mahabir et al. 2020; Sakal et al. 2021), and in Israel, PTSD MC license holders reached 13%, representing almost 20,000 patient (Aviram 2025). In all of these registries, patients primarily use inhaled MC, and not CBMs, which are usually confined to clinical trials. Given challenges in conducting RCTs, including issues of blinding and patenting, Real World Evidence (RWE), though not considered the gold standard, should still inform clinical decisions, especially for patients who have exhausted conventional therapies.

Therefore, the aim of this review is to systematically assess the current literature on CBMs and MC for PTSD symptom management and to evaluate their potential benefits and safety profiles. Specifically, our main research question was: What is the current state of academic evidence regarding the efficacy, effectiveness, and safety of CBMs and MC for PTSD?

## Materials and methods

This scoping review was conducted on December 24 2024, following the PRISMA-ScR checklist (Tricco et al. 2018) (displayed in appendix). The process included defining a research question, systematic database searching, study selection, data charting, and synthesis.

The review design was developed 'a priori' to data collection initiation but was not registered in PROSPERO prior to initiation. No meta-analysis was performed due to heterogeneity of reporting tools, scales and methods.

### Eligibility criteria

Included studies involved patients with PTSD diagnosis. Diagnosis was confirmed in RCTs by acceptable tools. For observational studies and registries, the diagnosis was based on the prescribing physician diagnosis, prior to inclusion in the registries.

Interventions included CBMs or MC for PTSD symptom management, regardless of outcome measures. Eligible designs included RCTs, cohort studies, case series, and observational studies. Synthetic cannabinoids (e.g., dronabinol, nabilone, Nabiximol, cannabidiol, ajulemic acid, PF-04457845, levonantradol) and cannabis extracts were included.

### Information sources and search strategy

We systematically searched PubMed, Cochrane Library, PsycINFO, Google Scholar, and ClinicalTrials.gov without date restrictions, limited to English-language articles. Search terms were grouped to cover the PICO (i.e., Population, Intervention, Comparator, Outcome) requirements under two main domains: (1) cannabis-based interventions (e.g., “cannabis”, “medical marijuana”, “THC”, “CBD”, “dronabinol”, “nabilone”, “cannabinoids”, “tetrahydrocannabinol”, “cannabis-based medicine”) (i.e., intervention and comparator) and (2) PTSD-related terms (“PTSD”, “post-traumatic stress disorder”) (i.e., population and outcome). Boolean operators (“AND”, “OR”) were used to combine terms, with syntax tailored per database. ClinicalTrials.gov was also searched for unpublished trials (i.e., grey literature), which were reported separately.

### Exclusion criteria

Studies were excluded if they did not include a PTSD population or if the study population was explicitly described by the authors as having cannabis misuse or cannabis use disorder (CUD), defined as non-medical cannabis use without a healthcare provider prescription; were case reports; were reviews, protocols, commentaries, or editorials; were qualitative-only studies; or if PTSD was a subpopulation not reported separately.

### Study selection

**Fig. 1:**
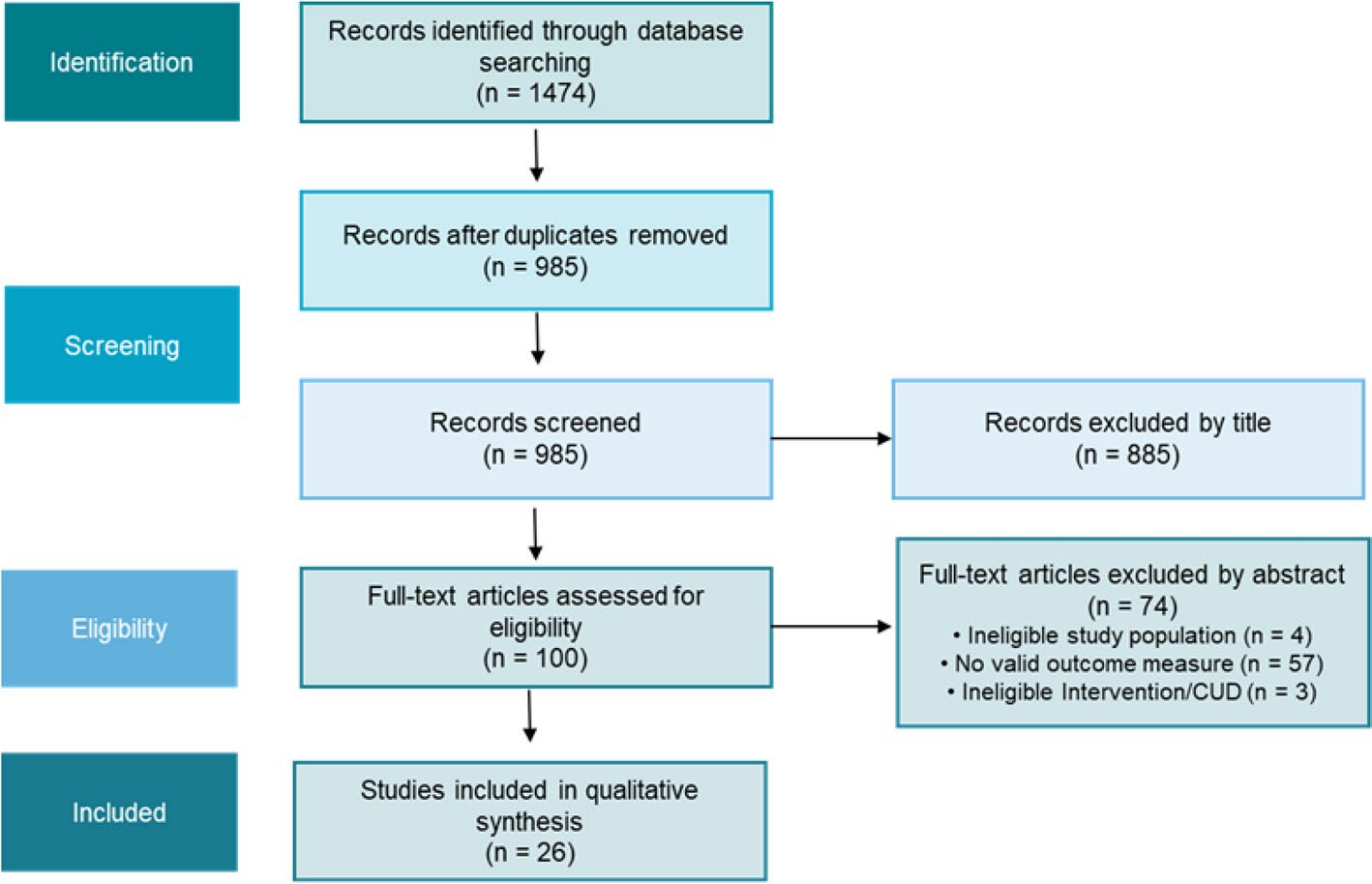
PRISMA flow diagram. CUD, cannabis use disorder

Two reviewers (JA and RB) independently screened (i.e., data selection and extraction were performed in duplicate) titles and abstracts. Duplicates were removed. Inclusion required studies on PTSD with clinically relevant outcomes. Full texts were then reviewed. Discrepancies were resolved by consensus. Figure 1 displays the PRISMA flow diagram for this scoping review.

### Data charting and synthesis

Extracted data included: study design, population size, intervention type, dosing, outcomes, route of administration, participant demographics, and prior cannabis experience. Adverse events (AEs) were categorized by type and frequency.

### Quality and risk of bias assessment

RCTs were assessed using the Jadad scale for quality and risk of bias (RoB), the majority showed moderate quality of evidence (Jadad et al. 1996) (Supplementary Table 3). Observational studies were assessed using the Newcastle–Ottawa Scale (NOS) (Luchini et al. 2017) (Supplementary Table 4). All four authors assessed the scores of the included studies. Discrepancies were resolved by consensus.

### Outcomes

Primary PTSD-related outcomes included changes in CAPS-5 (Clinician-Administered PTSD Scale for DSM-5), PCL-5 (PTSD Checklist for DSM-5), SF-MISS (Short Form Mississippi PTSD Scale), VAMS (Visual Analog Mood Scale), PROMIS-29 (Patient-Reported Outcomes Measurement Information System-29), QoLS (Quality of Life Scale), and CGI (Clinical Global Impression), among others. Some studies also employed physiological and imaging measures.

### Role of the funding source

The funder management (Syqe Medical) had no role in study design, data collection, analysis, or manuscript preparation.

## Results

### Selection of sources of evidence

The flow chart of studies through selection process is presented in Fig. 1. Out of 1,474 identified titles, 985 remained after duplicates were removed. Title and abstract screening excluded 959 studies. After full-text review, 26 studies met inclusion criteria, while 74 studies were excluded. The reasons for exclusion are summarized in Fig. 1 and detailed in Supplementary Table 1.

### Characteristics of sources of evidence

Of the 26 included studies (Jetly et al. 2015; ClinicalTrials.com. 2023; Vaddiparti et al. 2023; Stack et al. 2023; Pillai et al. 2022; Sznitman et al. 2022; Bonn-miller et al. 2022; Chan et al. et al. 2017; Roitman et al. 2014; Nacasch et al. 2023; Meakin et al. 2020; Lafrance et al. 2020; Zabik et al. 2024; Elms et al. 2019; Smith et al. 2017; Wilkinson et al. 2015; Greer et al. 2014; Cameron et al. 2014; Fraser 2009; Zabik et al. 2023; Walsh et al. 2023; Bolsoni et al. 2022; Bolsoni et al. 2022; Bonn-Miller et al. 2021; Sultan and Madiedo 2024; Lynskey et al. 2024), 7 were RCTs (Supplementary Tables 2 and 3), 9 were prospective observational trials (Supplementary Tables 4 and 5), 9 were retrospective observational trials (Supplementary Tables 6 and 7) and one was an RCT with unpublished results published in clinicaltrials.gov (described in the main text).

### RCTs: summary

Seven RCTs investigated CBMs/MC efficacy and safety in PTSD.

### RCTs: administration and exposure

Three RCTs used oral THC, two oral CBD, and two inhaled cannabis (via smoking or vaporization of full-spectrum formulations). Exposure durations ranged from a single cannabinoid session with 2 h follow-up to multi-administration trials for up to 7 weeks follow-up.

Dosage regimens in the RCTs varied widely. Oral THC (dronabinol) trials used fixed daily doses of 5 mg or 10 mg (Zabik et al. 2024) or a single 7.5 mg dose administrated in single session (Zabik et al. 2023), while the Nabilone RCT titrated from 0.5 mg up to 3 mg daily for 7 consecutive weeks (Jetly et al. 2015). Two RCTs administered oral CBD as a single session at a dose of 300 mg (Bolsoni et al. 2022; Bolsoni et al. 2022). In the inhaled MC trials, conducted in two sequential 3-week stages, participants self-titrated within protocol limits: one allowed ad libitum use of vaporized cannabis, and another permitted smoking up to 1.8 g/day of cannabis flower with ~ 12% THC (providing a theoretical maximum of ~ 216 mg THC per day].

### RCTs: effectiveness outcomes

Three RCTs reported symptom reductions (Jetly et al. 2015; Walsh et al. 2023; Bonn-Miller et al. 2021), but only one showed statistically significant benefit over placebo in CAPS score (Jetly et al. 2015). Two studies showed dose- and time-dependent increases in corticolimbic activation on fMRI with low-dose and high-dose THC (Zabik et al. 2023, 2024), indicating significant positive effects on fear extinction and renewal, and two oral CBD trials showed significant behavioral improvement of diminished trauma recall (Bolsoni et al. 2022; Bolsoni et al. 2022). In the abovementioned study, demonstrating a significant superiority of the treatment on placebo for the nightmares score of CAPS-5 (Jetly et al. 2015), it was a small crossover trial with Nabilone (synthetic THC) (*N =* 10, 0% dropout); the Nabilone arm showed a greater decrease in nightmare severity (CAPS recurring-dream item −3.6 points) compared to placebo (−1.0), a significant between-group difference. In contrast, the largest RCT (Bonn-Miller et al. 2021) (*N =* 80, 8% dropout) compared three smoked MC preparations (THC-dominant, CBD-dominant, balanced THC:CBD) to placebo over 3 weeks and found that all groups, including placebo, had moderate CAPS-5 symptom improvements (mean reductions ~ 8–15 points) with no significant differences between cannabis and placebo. Notably, the effect was greatest within the THC-rich arm, but with no superiority over placebo. In this study, the authors suggested that these findings are likely due to small sample size, unmasking of blinding due to patients' previous experience with cannabis or other reasons. Similarly, Walsh et al., (2023) (*N =* 6, 17% dropout) reported ~ 8-point CAPS-5 and a significant within-group PCL-5 reduction after 3 weeks of ad libitum vaporized MC (Walsh et al. 2023), but the sample (5 completers) was too small for between-group analysis. Two RCTs focusing on fear extinction mechanisms (Zabik et al. 2023; *N =* 86, 17% dropout and Zabik et al. 2024; *N =* 44, 18% dropout) did not measure clinical symptom change as the primary outcome; instead, they found that acute oral THC (7.5 mg) modulated neurobiological responses during extinction learning (e.g., increased vmPFC and amygdala activation) (Zabik et al. 2023), and that a 5–10 mg THC dose significantly reduced fear renewal in a laboratory paradigm (Zabik et al. 2024). However, neither of these trials observed a significant improvement in PTSD symptom scores or subjective fear ratings. Two small trials of oral CBD (*N =* 33 in each, dropout not reported) likewise did not significantly improve overall PTSD symptoms (Bolsoni et al. 2022; Bolsoni et al. 2022). Instead, CBD (300 mg single dose) significantly attenuated the increase in a post-recall cognitive impairment score on a Visual Analog Mood Scale (VAMS) compared to placebo, an effect that persisted one week after the trauma cue exposure. No significant effects of CBD were seen on anxiety (STAI-state) or physiological arousal (heart rate, blood pressure, cortisol) during trauma recall in these studies. See Supplementary Table 2 for a summary of RCT characteristics and outcomes.

### RCTs: unpublished RCT results

One unpublished RCT (ClinicalTrials.gov NCT05132699) evaluated CBD (adjunct to trauma-focused psychotherapy) versus placebo. It reported no significant differences between CBD and placebo arms in CAPS-5 or PCL-5 outcomes, and high rates of AEs in both arms (91% of CBD-treated vs 70% of placebo participants) (ClinicalTrials.com 2023). These findings are preliminary given the small sample (actual *N =* 21) and lack of peer-reviewed publication.

### RCTs: dropouts and risk of bias

Attrition ranged 0–17%. Risks of bias stemmed from lack of information on background PTSD treatment, prior cannabis use, or verification of abstinence/washout periods. Blinding may have been compromised by characteristic odor and psychoactive effects of cannabis, potentially leading participants to guess their assignment.

### RCTs: safety and tolerability

Only 2 of 7 RCTs (Jetly et al. 2015; Bonn-Miller et al. 2021) systematically reported AEs. The reported AEs were mostly mild-to-moderate. In the Bonn-Miller et al., (2021) trial, common treatment-related AEs included dry mouth (~ 15%), nausea (~ 10%), dizziness (~ 10%), and somnolence (~ 15%) (Bonn-Miller et al. 2021). Transient anxiety was noted (up to 17% in the high-THC group), and there were a few cases of paranoia or hallucinations (~ 5–10%) at THC containing doses. Upper respiratory tract infections (≈10%) and throat irritation (≈9%) were also observed, likely related to smoking/vaporization. Jetly et al., (2015) reported dry mouth (60%) and headache (40%) with Nabilone (Jetly et al. 2015). No serious AEs were reported in any RCT. Neuropsychiatric side effects (e.g., anxiety, irritability, paranoia) tended to be more common with THC-rich formulations, whereas CBD was well tolerated with no specific AEs reported in those trials.

### RCTs: dropouts due to AEs or inefficacy

Bonn-Miller et al., (2021) reported 8.4% attrition due to AEs (Bonn-Miller et al. 2021), none of the other RCTs clearly reported dropout reasons.

### Observational studies (prospective and retrospective): summary

A total of 9 prospective and 9 retrospective observational studies were included in the final analysis.

### Observational studies: dosing and administration

Cannabinoid dosing and administration in the observational studies were highly heterogeneous. Most prospective studies involved inhaled MC flower, typically with high Δ⁹-THC content (e.g., ~ 20% THC per product) or predominantly THC-dominant strains under real-world use. In some cohorts, patients combined inhalation with oral cannabis oils (approximately one-third of cases), whereas a smaller fraction exclusively used oral MC formulations. One observational study provided a fixed oral dose of 5 mg Δ⁹-THC daily via Dronabinol, but most “real-world” studies allowed patients to self-titrate their intake. The diversity of cannabinoid products was notable: many patients used full-spectrum botanical cannabis via smoking or vaporization, others used standardized extracts or synthetic THC, with THC concentrations varying widely. Doses were not standardized and often reported only as patient-reported use or as total dispensed amount, leading to a broad range of consumption (from a few milligrams of THC per day up to hundreds of milligrams in heavy users). This lack of standardization reflects real-world practice but complicates comparisons across studies and with RCTs.

### Observational studies: effectiveness

Among the 18 observational studies (9 prospective, 9 retrospective), 16 reported statistically significant or clinically meaningful improvements in at least one core PTSD outcome. In the 9 prospective studies, 8 reported significant symptom improvement, and in the 9 retrospective studies, 8 reported significant or marked improvement. Improvements were observed particularly in nightmares, hyperarousal, sleep quality, anxiety, and overall functioning.

For example, in prospective cohorts using validated PTSD scales (PCL-C, PCL-5, or CAPS-5), the magnitude of symptom reduction ranged from approximately −9 to −21 points on the PCL or CAPS scales over follow-up periods of 1 to 12 months, corresponding to changes typically considered moderate-to-large and clinically meaningful. Specifically, Sultan et al., (2024) (Sultan and Madiedo 2024) reported reductions of −13.0 at 3 months and −16.8 at 6 months on the PCL-C; Sakal et al., (2021) (Sakal et al. 2021) reported a −11.0 point reduction at 3 months; Vaddiparti et al., (2023) (Vaddiparti et al. 2023) reported −19.3 at 30 days and −20.6 at 70 days on the PCL-5; Roitman et al., (2014) reported a −16.0 point reduction on CAPS-5 over 3 weeks, alongside reduction in hyperarousal; and Bonn-Miller et al., (2022) (Bonn-Miller et al. 2022) observed a significantly greater decline in CAPS-5 scores among MC users compared with controls (−9.58 vs. −5.7 over 12 months).

Across retrospective studies, similarly large improvements were reported, including reductions of −14.7 to −15.9 points on the PCL-C (Elms et al. 2019; Cameron et al. 2014), and high proportions of patients reporting marked or near-complete symptom remission (e.g., 73% in Meakin et al., (2020) (67]; > 90% improvement in intrusions, flashbacks, irritability, and anxiety in Lafrance et al., (2020) (Lafrance et al. 2020); and a > 75% reduction on CAPS in a highly selected cohort, which also mentioned improvements in discrete PTSD symptom domains, including CAPS Criterion C (avoidance/numbing) and Criterion D (hyperarousal) (Greer et al. 2014)). Studies focusing specifically on sleep-related outcomes consistently reported substantial reductions in nightmare frequency and improvements in PSQI or insomnia-related measures, with 72%−97% of patients reporting meaningful benefit in some cohorts (Sznitman et al. 2022; Nacasch et al. 2023; Fraser 2009).

These improvements were often accompanied by increased quality of life and reductions in concurrent medication use (e.g., antidepressants, hypnotics). However, outcome measures varied across studies, and most were uncontrolled, relying on patient-reported symptoms without blinding or placebo groups. Additionally, baseline differences in PTSD severity, prior cannabis experience, and access to psychotherapy complicate causal attribution. Moreover, effect sizes were rarely reported in standardized form, precluding formal quantitative synthesis. Despite these limitations, the consistency of direction and the magnitude of symptom change across multiple large cohorts suggest potential real-world effectiveness in the PTSD population.

### Observational studies: safety

Adverse event reporting in observational studies was heterogeneous and often incomplete, but most studies described mild-to-moderate AEs, particularly with inhaled THC-dominant products. Commonly reported AEs included dry mouth (up to 25%), sedation (10–20%), dizziness (10–15%), and transient anxiety or paranoia (5–10%). A few retrospective cohorts also noted cognitive complaints, short-term memory impairment, and psychotomimetic effects, particularly with THC-rich strains or rapid dose escalation. However, serious AEs were rare, and no study reported persistent psychosis or hospitalization due to cannabinoid use. Notably, few studies systematically monitored AEs or used validated AE scales, and some relied entirely on voluntary self-reporting. In addition, long-term safety outcomes, such as dependency risk or tolerance development, were rarely assessed. As such, while the reported AE profile was generally benign, underreporting and lack of structured safety surveillance limit firm conclusions.

### Observational studies: dropouts and risk of bias

Dropouts ranged widely (6–68%). Reasons were often unclear or unreported. All observational studies had moderate to high risk of bias due to their non-randomized design, reliance on self-reported outcomes, high attrition, potential confounding (e.g., concurrent treatments), and lack of verified washout or control for prior cannabis use.

### Funding

Of the published RCTs, 85.7% were government-funded, while 14.3% (*n =* 1) were funded by industry. Among the prospective studies, 33.3% were either unfunded or did not report funding, 22.2% were industry-funded, and the remaining studies were supported by academic, philanthropic, or government sources (*n =* 1 each). In the retrospective studies, 55.6% were unfunded or did not report funding, while the rest received support from industry, academic, philanthropic, or government sources (*n =* 1 each).

### Critical appraisal

Methodological quality of RCTs was assessed with the Jadad scale (Supplementary Table 8), while observational studies were assessed with NOS (Supplementary Table 9). Common methodological limitations across studies included inadequate blinding, incomplete reporting of attrition and dropout reasons, high variability in baseline PTSD severity and access to psychotherapy, and limited use of intent-to-treat analyses. In cannabinoids studies, psychoactive side effects (e.g., acute intoxication) may unmask group assignment and introduce performance bias.

### Unpublished and registered trials from ClinicalTrials.gov

A total of 13 studies were identified through ClinicalTrials.gov entries for full report review. Of these, nine studies had no publicly available results (Supplementary Table 10). These included trials evaluating THC/CBD combinations, synthetic cannabinoids (e.g., dronabinol, nabilone), and oral CBD as adjuncts to psychotherapy or pharmacotherapy. Several trials were withdrawn prior to enrollment or terminated early due to recruitment challenges, protocol revisions, or funding issues (e.g., NCT03251326, NCT02039408, NCT03658623). These unpublished or incomplete studies represent potential sources of publication bias, as their absence may skew the literature toward positive findings.

In contrast, four registered trials did result in published findings (Supplementary Table 11). These included the majority of RCTs analyzed in this review, reflecting successful completion and outcome dissemination. Nevertheless, the lag between trial completion and publication, along with variability in reporting practices, limits the completeness of the available evidence.

## Discussion

Evidence from existing RCTs suggests that cannabinoids are not effective for PTSD symptom reduction. Nonetheless, clinical use of MC for PTSD is increasing worldwide (Aviram 2025). Our review, encompassing 26 studies involving 3,598 PTSD patients, shows that only one small RCT demonstrated superiority over placebo, whereas the remaining RCTs did not demonstrate significant between-group clinical benefit. Therefore, current evidence does not support clinical use of cannabinoids for PTSD, and further adequately powered, well-controlled RCTs are required.

Specifically, the seven included RCTs provided a variable picture. Only one RCT (*n =* 10) demonstrated a statistically significant improvement on core-related PTSD symptomology (nightmare frequency) with oral THC over placebo (Jetly et al. 2015). One particular RCT, which was the only one utilizing smoked botanical MC, rather than CBMs (excluding the underpowered study (Walsh et al. 2023)) require further discussion (Bonn-Miller et al. 2021), as its findings of no significant differences in CAPS-5 or PCL-5 symptom reductions between cannabinoid and placebo conditions is assumed to be related mainly to a very large effect size for the placebo arm. As the authors suggested, this may be a byproduct of blinding failure and small sample size per group (*n =* 20). It may also be speculated, that as all patients had previous cannabis experience, it was impossible to blind them from the arm they were allocated to, as was evident by their correct guess of being allocated to the THC containing arms. An unpublished 2020 RCT of oral CBD likewise showed no difference from placebo in PTSD symptom scores (ClinicalTrials.com 2023). Mentionable, CBD preparations are probably less likely to be effective, as real-world experience and trial-and-error process of patients, most commonly led them to select THC-rich products (Sultan and Madiedo 2024; Lynskey et al. 2024). Notably, exposure duration in these RCTs was extremely short, from single administration of 120 min to a maximum of 7 weeks, making long-term safety unverified.

Nonetheless, although not directly assessing PTSD symptomology, but related to other aspects of PTSD, other RCTs reported on therapeutic potential via fMRI indications for superior fear extinction and renewal (Zabik et al. 2023, 2024) and diminished trauma recall (Bolsoni et al. 2022; Bolsoni et al. 2022), superior to placebo.

Taken together, out of the three RCTs that assessed PTSD symptoms by CAPS-5, one demonstrated superiority, while in two, CBMs produced no significant difference in PTSD symptoms from the placebo arm, although this null finding may have been due to methodological flaws. Robust evidence of superior efficacy over placebo is still lacking. This underscores the need for larger, high-quality trials and partly explains the prevailing caution in clinical guidelines. Mechanistic outcomes related to fear extinction, fear renewal, or trauma recall may justify further investigation, but they should not be interpreted as evidence of clinical efficacy for PTSD symptom reduction.

In contrast, observational studies consistently suggest potential therapeutic benefits, with mainly MC, under medical supervision. However, the inherent limitations of real-world prospective and retrospective observational designs necessitate caution. While such studies provide a valuable signal of potential effectiveness, their findings must be interpreted carefully within the context of these methodological constraints.

Notably, the multiple observational data may be considered important, especially when considering the potential to assess long-term safety. Thus, across the included studies, exposure durations ranged from 15 days of multiple administrations per day to up to three years, and reported AEs were generally mild. However, because these studies were mostly uncontrolled and at moderate-to-high risk of bias, they cannot establish cannabinoid effectiveness for PTSD symptom reduction. Notably, these studies, unlike most included RCTs, utilized botanical MC, which may explain the discrepancy between the study design results. Nonetheless, due to the methodological limitations of observational designs, the current study minimized the contribution of these studies to the study final conclusions.

Considering the abovementioned, the current VA/DoD guideline (Lang et al. 2024) strongly advises against MC for PTSD, citing limited efficacy evidence and safety concerns. However, most of their concerns stem from the papers they cited from studies on recreational rather than MC use, traumatic brain injury, and unpublished conference abstracts. Therefore, those risks should not be generalized to MC as recreational cannabis properties are much more potent and unregulated (Mehmedic et al. 2010). Nonetheless, this finding supports a requirement for stricter clinical supervision, especially for AEs related to psychosis, whenever MC treatment is administered.

Currently, first-line treatments for PTSD include trauma-focused psychotherapies, such as cognitive behavioral therapy (CBT), prolonged exposure, and cognitive processing therapy. Evidence suggests military-related PTSD may be more treatment-resistant than civilian trauma (Bisson et al. 2013), with high non-completion rates for psychotherapy (12–48%) alongside modest pharmacotherapy efficacy (Steenkamp et al. 2020), highlighting the interest in alternative interventions. According to the updated clinical guidelines (Lang et al. 2024), next step should involve pharmaceutical treatment with SSRIs or SNRIs, like Paroxetine, Sertaline or Venlafaxine. Notably, a meta-analysis, with study samples ranging from 19 to 188 patients per arm demonstrated that SSRIs yielded small symptom reductions effect size based on CAPS-5 (standardized mean difference of −0.23, with 95% CI of −0.33 to −0.12). Of the 21 RCTs included, 10 demonstrated statistically significant superiority over placebo, 11 showed no significant difference, with notable AEs in PTSD population (Hoskins et al. 2015). Reported AEs included photophobia, blurry vision, tremor, weight gain affecting up to 17% of participants. Additionally, rates exceeding 10% were observed for dry mouth, asthenia, diarrhea, abnormal ejaculation, impotence, nausea, and somnolence.

In the three CBMs RCTs that did investigated PTSD symptoms via CAPS-5, one demonstrated significant reductions in nightmare frequency compared to placebo (Jetly et al. 2015) and two were not statistically superior to placebo (Walsh et al. 2023; Bonn-Miller et al. 2021). Given the lack of consistent superiority over placebo in RCTs and the absence of regulatory approval for PTSD, the current evidence does not support recommending CBMs or MC as PTSD treatments. Future trials should determine whether specific cannabinoid formulations, doses, routes of administration, or patient subgroups have clinically meaningful effects.

Comorbid chronic pain is common among patients with PTSD (Sharp and Harvey 2001) and may influence treatment seeking, symptom burden, and interpretation of cannabis-related outcomes (Asmundson et al. 2002; Kind et al. 2019). However, this review did not evaluate cannabinoids for chronic pain in PTSD populations and did not compare cannabinoids with other pharmacological or non-pharmacological PTSD treatments. Therefore, no recommendation can be made regarding cannabinoid use for PTSD patients with comorbid chronic pain based on the present review.

In summary, this review incorporates more studies than prior reviews (1–14 studies) (Lang et al. 2024; Belendiuk et al. 2015; Kansagara et al. 2017; Steenkamp et al. 2017; Wilkinson et al. 2016; Rehman et al. 2021; CADTH 2019; Cowling and MacDougall 2019; Black et al. 2019; O'Neil et al. 2017; Rodas et al. 2024; Passie et al. 2012), providing a broader and updated synthesis of the evidence, including preliminary efficacy signals and a generally tolerable safety profile. Mentionable, prior reviews did not fully consider the inherent pharmacological complexity of CBMs and MC or the structural barriers to high-quality RCTs, including patentability of botanicals and blinding difficulties due to a distinctive effect, smell, and taste. These challenges amplify the need to utilize RWE into the clinical decision process.

This review is limited by the predominance of observational studies and heterogeneous methodologies. High risk of bias, small RCT sample sizes, poor blinding, outcome variability, outcomes and diagnosis self-report in observational studies, and inconsistent AE reporting which hinder causal inference. In addition, variability in CBM and MC formulations, dosing, and administration further complicates comparison. Many studies lacked standardized reporting on CBM and MC content and did not control for confounding factors like concurrent medications or prior cannabis use. Also, Follow-up periods were often short, and generalizability is limited by male-dominant military cohorts. Because women were underrepresented in the reviewed studies, it remains unclear whether female PTSD patients experience different efficacy or safety profiles with cannabinoids. Future research should include more female and non-veteran PTSD populations to improve the generalizability of findings. Overlap between study populations may also bias findings. In addition, publication bias is possible as some of the studies published in clinicaltrials.gov have been terminated, suspended or delayed and may lead to an overestimation of the treatment efficacy.

## Conclusions

Current RCT evidence does not support the clinical use of cannabinoids for PTSD treatment. Among seven published RCTs, only one small trial demonstrated superiority over placebo, whereas the remaining trials did not show significant between-group clinical benefit. Observational studies frequently reported symptom improvement, but these findings are limited by uncontrolled designs, self-reported outcomes, attrition, confounding, and moderate-to-high risk of bias. Mechanistic findings related to fear extinction, fear renewal, or trauma recall may inform future research but should not be interpreted as evidence of clinical efficacy. Rigorous, adequately powered RCTs with standardized cannabinoid formulations, validated PTSD outcomes, longer follow-up, and systematic adverse-event monitoring are required before cannabinoids can be recommended for PTSD.

## Supplementary Information

Supplementary Material 1.

## Data Availability

All data analyzed during this study are included in this published article and are publicly available. Supplementary materials including the processed datasets are available from the corresponding author upon reasonable request.

## Abbreviations

AE: Adverse Event
CBD: Cannabidiol
CB1: Cannabinoid Receptor Type 1
CBMs: Cannabis-Based Medicines
CAPS-5: Clinician-Administered PTSD Scale for DSM-5
CBT: Cognitive Behavioral Therapy
CGI: Clinical Global Impression
CNCP: Chronic Non-Cancer Pain
CUD: Cannabis Use Disorder
DEA: Drug Enforcement Agency
DSM-5: Diagnostic and Statistical Manual of Mental Disorders, Fifth Edition
FDA: Food and Drug Administration
fMRI: Functional Magnetic Resonance Imaging
MC: Medical Cannabis
NOS: Newcastle–Ottawa Scale
PCL-5: PTSD Checklist for DSM-5
PICO: Population, Intervention, Comparator, Outcome
PRISMA-ScR: Preferred Reporting Items for Systematic Reviews and Meta-Analyses Extension for Scoping Reviews
PROMIS-29: Patient-Reported Outcomes Measurement Information System-29
PTSD: Post-Traumatic Stress Disorder
RCT: Randomized Controlled Trial
RoB: Risk of Bias
RWE: Real-World Evidence
SF-MISS: Short Form Mississippi PTSD Scale
SNRI: Serotonin–Norepinephrine Reuptake Inhibitor
SSRI: Selective Serotonin Reuptake Inhibitor
STAI: State-Trait Anxiety Inventory
THC: Δ9-Tetrahydrocannabinol
US: United States
VA/DoD: United States Department of Veterans Affairs / Department of Defense
VAMS: Visual Analog Mood Scale
vmPFC: Ventromedial Prefrontal Cortex
WHO: World Health Organization
