# Supplementary material for: Efficacy, effectiveness and safety of medical cannabis in PTSD: a scoping review
Source: J Cannabis Res. 2026 May 29;8:89. doi: 10.1186/s42238-026-00451-7 (PMC13425960; doi:10.1186/s42238-026-00451-7)
Supplement: Supplementary file 1 — Supplementary Material 1. [file 42238_2026_451_MOESM1_ESM.docx]

**Appendixes**

**Supplementary Table 1: Summary of studies excluded from review with reason for exclusion**

**Supplementary Table 2: Summary of RCTs characteristics and efficacy results**

**Supplementary Table 3: Summary of RCTs safety outcomes**

**Supplementary Table 4: Summary of** **prospective observational studies characteristics and efficacy results**

**Supplementary Table 5: Summary of prospective observational studies safety outcomes**

**Supplementary Table 6: Summary of** **retrospective observational studies characteristics and efficacy results**

**Supplementary Table 7: Summary of retrospective observational studies safety outcomes**

**Supplementary Table 8: Jadad quality scores for RCTs**

**Supplementary Table 9: Newcastle–Ottawa Scale for observational studies evaluation**

**Supplemental Table 10: Summary of Studies Identified from ClinicalTrials.gov without published Results**

**Supplemental Table 11: Summary of Studies Identified from ClinicalTrials.gov with Peer-reviewed Published Results**

**PRISMA 2020 Checklist**

**Supplementary Table 1:** **Summary of studies excluded from review with reason for exclusion**

| **Index** | **First author, year** | **Title** | **Reason for exclusion** |
| --- | --- | --- | --- |
| 1 | Wolfgang A, 2023 | Cannabis and Cannabinoids for Pain and Posttraumatic Stress Disorder in Military Personnel and Veterans | No valid outcome measure - author’s perspective, interpretation, or commentary on a topic. |
| 2 | Ragnhildstveit A, 2023 | Cannabis-assisted psychotherapy for complex dissociative posttraumatic stress disorder: A case report | No valid outcome measure – case report checks the effectiveness of psychotherapy. |
| 3 | Ragen B, 2015 | Investigational drugs under development for the treatment of PTSD | No valid outcome measure - author’s perspective, interpretation, or commentary on a topic. |
| 4 | Bonn-Miller M, 2014 | Using cannabis to help you sleep: Heightened frequency of medical cannabis use among those with PTSD. | Ineligible study population – Cannabis users with probably PTSD diagnosis |
| 5 | Bonn-Miller M, 2014 | Self-reported cannabis use characteristics, patterns and helpfulness among medical cannabis users. | Ineligible study population – Cannabis users with probably PTSD diagnosis |
| 6 | Bonn-Miller M, 2011 | Posttraumatic stress, difficulties in emotion regulation, and coping-oriented marijuana use. | No valid outcome measure – Checking emotional regulation with now other valid outcome. |
| 7 | Bonn-Miller M, 2011 | Cannabis use among military veterans after residential treatment for posttraumatic stress disorder. | CUD population- Military veterans in rehabilitation center |
| 8 | Straud C, 2024 | Enhancing massed prolonged exposure with cannabidiol to improve posttraumatic stress disorder: Design and methodology of a pilot randomized clinical trial | No valid outcome measure – Pilot study with no result reported. |
| 9 | Straud C, 2023 | Combining Cannabidiol with Prolonged Exposure Therapy for PTSD: Design and Methodology of a Pilot Randomized Clinical Trial | No valid outcome measure – Poster describing methodology. |
| 10 | Calhoun P, 2000 | Drug use and validity of substance use self-reports in veterans seeking help for posttraumatic stress disorder | No valid outcome measure – Only substance use increase reported. |
| 11 | Ciesluk B, 2024 | Corrigendum: Cannabis use in the UK: A quantitative comparison of individual differences in medical and recreational cannabis users. | CUD population- Difference between recreational and medical cannabis users |
| 12 | Cougle J, 2011 | Posttraumatic stress disorder and cannabis use in a nationally representative sample | No valid outcome measure – Epidemiology study to inspect chance for cannabis use. |
| 13 | Davis A, 2016 | Factors associated with having a medical marijuana card among Veterans with recent substance use in VA outpatient treatment. | No valid outcome measure – Epidemiology study to evaluate the prevalence of cannabis use among veterans. |
| 14 | De Aquino J, 2020 | Impact of cannabis on non-medical opioid use and symptoms of posttraumatic stress disorder: A nationwide longitudinal VA study. | No valid outcome measure – Epidemiology study to evaluate the prevalence of opioids use. |
| 15 | DeGrace S, 2023 | Do trauma cue exposure and/or PTSD symptom severity intensify selective approach bias toward cannabis cues in regular cannabis users with trauma histories? | No valid outcome measure – Outcome measure out of scope of this review. |
| 16 | Earleywine M, 2014 | Marijuana, expectancies, and post-traumatic stress symptoms: A preliminary investigation. | No valid outcome measure – Outcome measure out of scope of this review: study investigate participants expectation from cannabis. |
| 17 | Elms L, 2020 | Cannabidiol in the treatment of post-traumatic stress disorder: A case series': Reply | No valid outcome measure – Authors reply. |
| 18 | Farrelly K, 2024 | Indirect associations between PTSD symptoms and cannabis problems in young adults: The unique roles of cannabis coping motives and medicinal use orientation. | No valid outcome measure – Outcome measure out of scope of this review: study investigate cannabis orientation and trauma-related coping |
| 19 | Fitzke R, 2022 | Co-use of tobacco products and cannabis among veterans: A preliminary investigation of prevalence and associations with mental health outcomes | No valid outcome measure – Outcome measure out of scope of this review: Check the co-use of tabaco and cannabis |
| 20 | Gendy M, 2023 | Prevalence of cannabis use disorder among individuals using medical cannabis at admission to inpatient treatment for substance use disorders. | No valid outcome measure - Classification of cannabis use population by CUD chareacteristics. |
| 21 | Gentes E, 2016 | Prevalence and correlates of cannabis use in an outpatient VA posttraumatic stress disorder clinic | No valid outcome measure – Outcome measure out of scope of this review: screening for cannabis use |
| 22 | Goodrum N, 2022 | Interpersonal violence, PTSD, and substance use types among women receiving substance use treatment | Not valid population: all women in risk for substance use disorder, also were examinate for PTSD |
| 23 | Grant S, 2016 | Associations of posttraumatic stress disorder symptoms with marijuana and synthetic cannabis use among young adult U.S. veterans: A pilot investigation. | Not valid population: sample of veterans screened for PTSD and cannabis use |
| 24 | Hale A, 2021 | Residential PTSD treatment outcomes during cognitive processing therapy for veterans with and without recent histories of cannabis use. | No valid outcome measure – Outcome measure out of scope of this review: check the result of the cognitive processing therapy |
| 25 | Hinojosa C, 2023 | Associations of alcohol and cannabis use with change in posttraumatic stress disorder and depression symptoms over time in recently trauma-exposed individuals. | No valid outcome measure – Outcome measure out of scope of this review: Check the co-use of alcohol and cannabis |
| 26 | Krediet E, 2020 | Experiences with medical cannabis in the treatment of veterans with PTSD: Results from a focus group discussion. | No valid outcome measure – Quality study to investigate patients Luke opinion |
| 27 | Krumm B, 2016 | Cannabis for posttraumatic stress disorder: A neurobiological approach to treatment | No valid outcome measure - author’s perspective, interpretation, or commentary on a topic. |
| 28 | Lake S, 2020 | Does cannabis use modify the effect of post-traumatic stress disorder on severe depression and suicidal ideation? Evidence from a population-based cross-sectional study of Canadians. | No valid outcome measure – Search for major depression and suicidal ideation |
| 29 | Leen N, 2022 | The role of the endocannabinoids 2-AG and anandamide in clinical symptoms and treatment outcome in veterans with PTSD. | No valid outcome measure – Hormone level prediction |
| 30 | Lesia M, 2017 | Impact of Cannabis Use on Treatment Outcomes among Adults Receiving Cognitive-Behavioral Treatment for PTSD and Substance Use Disorders | No valid outcome measure – Association between PTSD and substance use disorder |
| 31 | Lisa F, 2018 | A treatment development study of a cognitive and mindfulness-based therapy for adolescents with co-occurring post-traumatic stress and substance use disorder | No valid outcome measure – Association between cognitive treatment for PTSD and cannabis use reduction |
| 32 | Luke N, 2023 | Australian clinical feasibility considerations for treatment of PTSD with cannabinoid-augmented exposure therapy | No valid outcome measure - author’s perspective, interpretation, or commentary on a topic. |
| 33 | Loflin M, 2019 | A cross-sectional examination of choice and behavior of veterans with access to free medicinal cannabis | No valid outcome measure – Association between PTSD and substance use disorder |
| 34 | Boden M, 2013 | Posttraumatic stress disorder and cannabis use characteristics among military veterans with cannabis dependence | No valid outcome measure – Association between Veterans with PTSD and substance use disorder |
| 35 | Telch M, 2022 | Use of Cannabidiol (CBD) oil in the treatment of PTSD: Study design and rationale for a placebo-controlled randomized clinical trial | No valid outcome measure – Description of future study with no result reported. |
| 36 | No authors listed, 2015 | Long-term Nabilone Use: A Review of the Clinical Effectiveness and Safety | No valid outcome measure - author’s perspective, interpretation, or commentary on a topic |
| 37 | Ouellette M, 2022 | Does cannabis use impact cognitive behavioral therapy outcomes for anxiety and related disorders? A preliminary examination. | No valid outcome measure – Association between cannabis use by PTSD patients and cognitive therapy outcome |
| 38 | Phillips K, 2024 | Psychiatric and substance use disorders among adults over age 50 who use cannabis: A matched cohort study using electronic health record data. | Invalid population – Retrospective inspection of population who reported cannabis use |
| 39 | Rehder K, 2019 | PTSD symptom severity, cannabis, and gender: A zero-inflated negative binomial regression model. | No valid outcome measure – Prediction of cannabis use by PTSD patients |
| 40 | Reyes-Velez J, 2022 | Characteristics of Canadian veterans reimbursed for cannabis for medical purposes: Life After Service Survey 2016. | Invalid population – Retrospective inspection of whole veteran population who reported medical cannabis use |
| 41 | Roepke S, 2023 | Treating nightmares in posttraumatic stress disorder with dronabinol: Study protocol of a multicenter randomized controlled study (THC PTSD trial). | No valid outcome measure – Description of future study with no result reported. |
| 42 | Romero-Sanchiz P, 2022 | Craving and emotional responses to trauma and cannabis cues in trauma-exposed cannabis users: Influence of PTSD symptom severity. | No valid outcome measure – Association between cannabis craving as result of PTSD symptoms |
| 43 | Ruba S, 2024 | Reasons for Use and Perceived Effects of Medical Cannabis: A Cross-Sectional Statewide Survey | Invalid population – Retrospective inspection of population who reported cannabis use |
| 44 | Saba S, 2024 | Pain and multi-morbidity among veterans: Theory-guided, data-driven, and narrative approaches. | No valid outcome measure – Machine learning model for recognizing predictors of pain in PTSD patients. |
| 45 | Short N, 2015 | Sleep quality, problematic cannabis use and posttraumatic stress symptoms among medical cannabis users. | Invalid population – Investigation of patients with Post-Traumatic stress symptoms |
| 46 | Stuyt E, 2020 | 'Cannabidiol in the treatment of post-traumatic stress disorder: A case series': Comment. | No valid outcome measure – author’s perspective, interpretation, or commentary on a topic. |
| 47 | Cowling T, 2019 | Nabilone for the Treatment of Post-Traumatic Stress Disorder: A Review of Clinical Effectiveness and Guidelines | No valid outcome measure – author’s perspective, interpretation, or commentary on a topic. |
| 48 | Torsten P, 2012 | Mitigation of post-traumatic stress symptoms by Cannabis resin: a review of the clinical and neurobiological evidence | No valid outcome measure – author’s perspective, interpretation, or commentary on a topic. |
| 49 | Tull M, 2016 | Marijuana dependence moderates the effect of posttraumatic stress disorder on trauma cue reactivity in substance dependent patients. | CUD population- Investigation of population with CUD, some of them with PTSD |
| 50 | Ueno L, 2024 | PTSD, cannabis use, and expected symptom relief: Gender-moderated mediational effects. | No valid outcome measure – Investigate association between cannabis use and expectation of symptoms relief. |
| 51 | Ursano R, 2009 | PTSD and traumatic stress from gene to community and bench to bedside | No valid outcome measure – Inspection of cannabinoid receptors in PTSD patients |
| 52 | Walukevich D, 2019 | Sexual minority women and Cannabis use: The serial impact of PTSD symptom severity and coping motives. | No valid outcome measure – Description of specific population of PTSD patient with no outcome analysis. |
| 53 | Wease T, 2022 | Cannabis use and posttraumatic stress disorder symptom severity among Californians. | No valid outcome measure – Analysis of association between CUD and PTSD symptoms severity among PTSD patients. |
| 54 | Williamson R, 2024 | Mapping the pathways between posttraumatic stress disorder, depression, and alcohol and cannabis use: A network analysis. | No valid outcome measure – Analysis of association between SUD and PTSD symptoms severity among PTSD patients |
| 55 | NCT00965809 | Add on Study on Î”9-THC Treatment for Posttraumatic Stress Disorders (PTSD) | No valid outcome measure – No results posted |
| 56 | NCT02069366 | Cannabinoid Control of Fear Extinction Neural Circuits in Post-traumatic Stress Disorder | No valid outcome measure – fMRI results |
| 57 | NCT02517424 | Evaluating Safety and Efficacy of Cannabis in Participants With Chronic Posttraumatic Stress Disorder | No valid outcome measure – No results posted |
| 58 | NCT02874898 | Short-Term Exposure for PTSD | No valid outcome measure – No results posted |
| 59 | NCT03008005 | Effects of Delta-9 Tetrahydrocannabinol (THC) on Retention of Memory for Fear Extinction Learning in PTSD: R61 Study | No valid outcome measure – fMRI results |
| 60 | NCT03248167 | Cannabidiol as a Treatment for AUD Comorbid With PTSD | No valid outcome measure – Alcohol use disorder reduction |
| 61 | NCT03251326 | Nabilone in Cannabis Users With PTSD | No valid outcome measure – No results posted |
| 62 | NCT04080427 | Effects of Delta9-tetrahydrocannabinol (THC) on Retention of Memory for Fear Extinction Learning in PTSD: R33 Study | No valid outcome measure – No results posted |
| 63 | NCT04197102 | Use of CBD Oil in the Treatment of Posttraumatic Stress Disorder | No valid outcome measure – No results posted |
| 64 | NCT04448808 | Treating Nightmares in Posttraumatic Stress Disorder with Dronabinol | No valid outcome measure – No results posted |
| 65 | NCT04550377 | Cannabidiol as a Treatment for PTSD and PTSD Comorbid With TBI | No valid outcome measure – No results posted |
| 66 | NCT04565028 | Functional Outcomes of Cannabis Use (FOCUS) in Veterans with Posttraumatic Stress Disorder | No valid outcome measure – No results posted |
| 67 | NCT04592159 | Study of the Safety and Efficacy of Nabiximols Oromucosal Spray Versus Placebo in Patients With Post-traumatic Stress Disorder | No valid outcome measure – No results posted |
| 68 | NCT04871269 | The Effect of Dronabinol on the Acquisition and Consolidation of Trauma-Associated Memories | No valid outcome measure – No results posted |
| 69 | NCT04965740 | Exploring Medically Perceived Benefits, Use and Interest in Psychedelics and Cannabinoids | No valid outcome measure – No results posted |
| 70 | NCT05269459 | Safety and Efficacy of Cannabidiol (CBD) for Symptoms of PTSD in Adults | No valid outcome measure – No results posted |
| 71 | NCT05579717 | Examining EAL for PTSD | No valid outcome measure – No results posted |
| 72 | NCT05892276 | Effects of CBD Oil on Memory Reconsolidation and Trauma-Related Symptoms | No valid outcome measure – No results posted |
| 73 | NCT06381180 | Warrior CARE: Cannabis Behavioral Health | No valid outcome measure – No results posted |
| 74 | NCT03518801 | Cannabidiol and Prolonged Exposure | No valid outcome measure – No results posted |

**Supplementary Table 2:** **Summary of RCTs characteristics and efficacy results**

| First author, year (CT.gov if applicable) | Design (duration) | N enrolled (N and % dropout) | Administration route | Intervention drug | Age (M, years) | Male (%) | Method of primary outcome | Method of secondary outcomes | Baseline  primary outcome | Previous cannabis experience | Funding (source) | WO pre study | Primary outcome change | Quality* |
| --- | --- | --- | --- | --- | --- | --- | --- | --- | --- | --- | --- | --- | --- | --- |
| Zabik, 2024 ^1^ (N/A) | Double-blind randomized,  parallel, placebo-controlled (1 week) | 44 (8; 18%) | Oral capsule | 1. 5 mg Dronabinol.  2. 10 mg Dronabinol. 3. Placebo capsule | 24.5 | 70% | Extinction recalls and fear renewal | Drug Effect Questionnaire, US expectancy ratings, Whole brain analysis and Regions of interest (ROIs) | N/A | N/A | National Institute of Mental Health (government), Mental Health Initiative in Stress and Trauma (academia) | negative on a seven-panel urine drug screen and alcohol breathalyzer | No effect of THC dose on fear (expectancy) ratings. Significant effect on fear renewal† | 4 |
| Zabik, 2023^2^ (N/A) | Double-blind randomized, parallel-group, placebo-controlled (120 minutes) | 86 (15; 17%) | Oral capsule | 1. 7.5 mg Dronabinol.  2. Dextrose capsule | 26.1 | 51% | fMRI to measure fear extinction | Skin conduction and expectancy scores | N/A | N/A | National Institute of Mental Health (government) | negative on a urine drug screen and breathalyzer | No effect of drug or trauma on US expectancy ratings was found. During early extinction learning, individuals with PTSD given THC had greater vmPFC activation than their TEC counterparts. During a test of the return of fear (i.e., renewal), HC and individuals with PTSD given THC had greater vmPFC activation compared to TEC. Individuals with PTSD given THC also had greater amygdala activation compared to those given PBO | 4 |
| Walsh, 2023^3^ (NCT02517424) | Double-blind randomized, crossover, placebo-controlled (3 weeks) (crossover stage not analyzed) | 6 (1; 17%) | Vaporized cannabis flowers (main components defined) | ad lib use 1. THC 10%: CBD <1%.  2. CBD 10%: THC 10%. (data combined due to small sample) 3. <1% THC and <1% CBD cannabis flower placebo | N/A | N/A | CAPS-5 | PCL-5 | 1+2. 39.0  3. N/A | N/A | Tilray (commercial) | N/A | 1+2. -8.33  3. N/A | 2 |
| Bolsoni, 2022^4^ (N/A) | Double-blind randomized, parallel-group, placebo-controlled (7 days) | 33 (N/A; N/A) | Oral CBD | 1.Powder dissolved in corn oil within capsules (300 mg). Before audio cue for trauma 2. Corn oil only in identical capsules | 33.2 | 24% | VAMS | PCL-5, BP, HR, Salivary cortisol and STAI-state | N/A | N/A | São Paulo Research Foundation (government), National Institute of Science and Technology for Translational Medicine (government) | N/A | The differences between measurements before and after recall were significantly† smaller with CBD than placebo for the cognitive impairment factor.  No effect of CBD was observed on anxiety, alertness and discomfort induced by the recall. | 4 |
| Bolsoni, 2022^5^ (N/A) | Double-blind randomized, parallel-group, placebo-controlled (7 days) | 33 (N/A; N/A) | Oral CBD | 1.Powder dissolved in corn oil within capsules (300 mg). Before audio cue for trauma 2. Corn oil only in identical capsules | 33.9 | 24% | VAMS | BP, HR, Salivary cortisol | N/A | N/A | São Paulo Research Foundation (government), National Institute of Science and Technology for Translational Medicine (government) | N/A | CBD significantly† attenuated the increase in the VAMS scale cognitive impairment factor scores, under the CBD’s effect, with this effect remaining 1 week after drug administration | 4 |
| Bonn-Miller, 2021 ^6^ (N/A) | Double-blind randomized, crossover, placebo-controlled (3 weeks) (crossover stage not analyzed) | 80 (6; 8%) | Smoked cannabis flowers (main components defined) | Up to 1.8 gr/day: 1. THC 12%: CBD <0.05.  2. CBD 11%: THC 0.5%. 3. THC 7.9%: CBD 8.1% 4. NIDA cannabis flower placebo | 44.9 | 90% | CAPS-5 | PCL-5, IDAS, IPF | 1. 36.6  2. 36.8  3. 38.0  4. 37.3 | Yes, for all | Colorado Department of Public Health and Environment (government), Multidisciplinary Association for Psychedelic Studies (MAPS) (non-profit 501(c)(3) organization) | N/A | 1. -15.2†  2. -8.4†  3. -8.5†  4. -13.1† | 4 |
| Jetly, 2015^7^ (N/A) | Double-blind randomized,  crossover, placebo-controlled (7 weeks) | 10 (0; 0%) | Oral | 1. Nabilone (initial daily dose 0.5 mg up to 3.0 mg) 2. Placebo | 43.6 | 100% | CAPS-5 Recurring and Distressing Dream Item | CGI-C, WBQ | N/A | N/A | Canadian Forces Surgeon General’s Health Research Program (government) | Negative for THC at screening | 1. -3.6∆  2. -1.0 | 4 |

BP, Blood Pressure; CAPS-5, Clinician-Administered PTSD Scale for DSM-5; CBD, Cannabidiol; D, day; EQ-5D-5L, EuroQol 5 Dimensions 5 Levels Questionnaire; fMRI, Functional Magnetic Resonance Imaging; HR, Heart Rate; GAD-7, Generalized Anxiety Disorder 7-Item Scale; GCI, Global Clinical Impression; IDAS, Inventory of Depression and Anxiety Symptoms; IES-R, Impact of Event Scale – Revised; IPAQ, International Physical Activity Questionnaire; IPF, Inventory of Psychosocial Functioning; ISI, Insomnia Severity Index; M, month; MC, medical cannabis; N/A, Not Applicable; NES, Negative Effects Scale; NFQ, Nijmegen Fatigue Questionnaire; NWAK, number of awakenings; PANAS, Positive and Negative Affect Schedule; PCL-5, PTSD Checklist for DSM-5; PCL-C, PTSD Checklist – Civilian Version; PGIC, Patient Global Impression of Change; PHQ-9, Patient Health Questionnaire-9; PSQI, Pittsburgh Sleep Quality Index; SOL, sleep onset latency; STAI, State-Trait Anxiety Inventory; SQS, Sleep Quality Scale; TEC, Traumatic Experiences Checklist; THC, Δ⁹-Tetrahydrocannabinol; TST, total sleep time; VAMS, Visual Analog Mood Scale; W, week; WAS, wake after sleep onset; WBQ, Well-Being Questionnaire; WO, washout; *, Quality based on Jadad scale, †, Significant improvement from baseline, ∆, Significant improvement compared to placebo.

**Supplementary Table 3:** **Summary of RCTs safety outcomes**

| **First author, year** | **Adverse events n/N (%) for intervention group/s all treatment related and unrelated reports** | **Adverse events n/N (%) for intervention group/s all treatment related and unrelated reports by SOC** |
| --- | --- | --- |
| Zabik, 2024 ^1^ | Not reported | Not reported |
| Zabik, 2023^2^ | Not reported | Not reported |
| Walsh, 2023^3^ | Not reported | Not reported |
| Bolsoni, 2022^4^ | Not reported | Not reported |
| Bolsoni, 2022^5^ | Not reported | Not reported |
| Bonn-Miller, 2021^6^* | THC-rich arm: Stage 1: Related: Tachycardia 1/20 (5.0%), Dry eye 2/20 (10.0%), Dry mouth 3/20 (15.0%), Nausea 2/20 (10.0%), Dizziness 2/20 (10.0%), Somnolence 3/20 (15.0%), Irritability 2/20 (10.0%), Anxiety 1/20 (5.0%), Hallucinations (visual) 1/20 (5.0%), Paranoia 1/20 (5.0%), Insomnia 1/20 (5.0%). Not related: Influenza 1/20 (5.0%), Upper respiratory tract infection 3/20 (15.0%), Back pain 2/20 (10.0%), Neck pain 1/20 (5.0%), Panic attack 1/20 (5.0%), Pollakiuria 1/20 (5.0%), Urine abnormality 1/20 (5.0%).  Stage 2 Related: Dry eye 1/29 (3.4%), Dry mouth 2/29 (6.9%), Nausea 3/29 (10.3%), Vomiting 3/29 (10.3%), Dizziness 2/29 (6.9%), Feeling abnormal 1/29 (3.4%), Anxiety 5/29 (17.2%), Insomnia 1/29 (3.4%), Paranoia 3/29 (10.3%). Not related: Upper respiratory tract infection 2/29 (6.9%), Back pain 2/29 (6.9%), Sinusitis 1/29 (3.4%), Throat irritation 2/29 (6.9%).  CBD-rich arm: Stage 1 Related: Dry mouth 1/20 (5.0%), Nausea 2/20 (10.0%), Anxiety 1/20 (5.0%), Insomnia 1/20 (5.0%), Irritability 1/20 (5.0%). Not related: Upper respiratory tract infection 2/20 (10.0%), Back pain 1/20 (5.0%), Throat irritation 4/20 (20.0%).  Stage 2 Related: Dry mouth 2/27 (7.4%), Anxiety 3/27 (11.1%), Insomnia 2/27 (7.4%), Irritability 1/27 (3.7%), Depression 1/27 (3.7%). Not related: Upper respiratory tract infection 4/27 (14.8%), Sinusitis 2/27 (7.4%), Back pain 2/27 (7.4%), Throat irritation 1/27 (3.7%)  THC:CBD balanced arm:  Stage 1 Related: Abdominal discomfort 1/20 (5.0%), Nausea 5/20 (25.0%), Vomiting 1/20 (5.0%), Dizziness 3/20 (15.0%), Tremor 1/20 (5.0%), Anxiety 2/20 (10.0%), Paranoia 4/20 (20.0%), Libido increased 1/20 (5.0%). Not related: Upper respiratory tract infection 1/20 (5.0%), Back pain 1/20 (5.0%), Throat irritation 3/20 (15.0%).  Stage 2 Related: Dry eye 1/18 (5.6%), Nausea 1/18 (5.6%), Vomiting 1/18 (5.6%), Dizziness 1/18 (5.6%), Feeling abnormal 1/18 (5.6%), Anxiety 2/18 (11.1%), Insomnia 1/18 (5.6%), Irritability 2/18 (11.1%). Not related: Upper respiratory tract infection 1/18 (5.6%), Back pain 3/18 (16.7%), Heat exhaustion 2/18 (11.1%) | THC-arm:  Stage 1 Related: Cardiac disorders 1/20 (5.0%)  Eye disorders 2/20 (10.0%), Gastrointestinal disorders 5/20 (25.0%), Nervous system disorders 6/20 (30.0%)  General disorders and administration site conditions 2/20 (10.0%), Psychiatric disorders 3/20 (15.0%) Not related: Respiratory, thoracic and mediastinal disorders 4/20 (20.0%), Musculoskeletal and connective tissue disorders 3/20 (15.0%), Psychiatric disorders 1/20 (5.0%), Renal and urinary disorders 2/20 (10.0%)  Stage 2 Related: Eye disorders 1/29 (3.4%)  Gastrointestinal disorders 8/29 (27.6%) Nervous system disorders 3/29 (10.3%), General disorders and administration site conditions 1/29 (3.4%), Psychiatric disorders 8/29 (27.6%), Respiratory, thoracic and mediastinal disorders 4/29 (13.8%) Musculoskeletal and connective tissue disorders 2/29 (6.9%), Infections and infestations 1/29 (3.4%). Not related: Respiratory, thoracic and mediastinal disorders 4/29 (13.8%)  Musculoskeletal and connective tissue disorders 2/29 (6.9%), Infections and infestations 1/29 (3.4%).  CBD-rich arm:  Stage 1 related: Gastrointestinal disorders 3/20 (15.0%) Psychiatric disorders 1/20 (5.0%)  Nervous system disorders 1/20 (5.0%)  General disorders and administration site conditions 1/20 (5.0%). Not related: Respiratory, thoracic and mediastinal disorders 5/27 (18.5%) Infections and infestations 2/27 (7.4%) Musculoskeletal and connective tissue disorders 2/27 (7.4%)  Stage 2 Related: Gastrointestinal disorders 2/27 (7.4%)  Psychiatric disorders 4/27 (14.8%) Nervous system disorders 2/27 (7.4%) General disorders and administration site conditions 1/27 (3.7%). Not related: Respiratory, thoracic and mediastinal disorders 5/27 (18.5%) Infections and infestations 2/27 (7.4%) Musculoskeletal and connective tissue disorders 2/27 (7.4%)  THC:CBD balanced arm:  Stage 1 Related: Gastrointestinal disorders 7/20 (35.0%), Nervous system disorders 4/20 (20.0%), Psychiatric disorders 7/20 (35.0%). Not related: Respiratory, thoracic and mediastinal disorders 4/20 (20.0%). Musculoskeletal and connective tissue disorders 1/20 (5.0%)  Stage 2 Related: Eye disorders 1/18 (5.6%)  Gastrointestinal disorders 2/18 (11.1%), Nervous system disorders 2/18 (11.1%), General disorders and administration site conditions 3/18 (16.7%), Psychiatric disorders 2/18 (11.1%)  Not related: Respiratory, thoracic and mediastinal disorders 1/18 (5.6%), Musculoskeletal and connective tissue disorders 3/18 (16.7%), Injury, poisoning and procedural complications 2/18 (11.1%) |
| Jetly, 2015^7^ | Dry mouth 6/10 (60%), Headache 4/10 (40%)- No additional comprehensive data provided | Gastrointestinal disorders 6/10 (60.0%)  Nervous system disorders 4/10 (40.0%) |

CBD, Cannabidiol; THC, Δ⁹-Tetrahydrocannabinol; SOC, System organ class.;*Data displayed for stage 1 of the study only as no efficacy analyses were based on stage 2.

**Supplementary Table 4:** **Summary of prospective observational studies characteristics and efficacy results**

| First author, year | Design (duration) | N enrolled (N and % dropout) | Administration route | Intervention drug | Control group | Age (M, years) | Male (%) | Method of primary outcome | Method of secondary outcomes | Baseline  primary outcome | Previous cannabis experience | Background treatment | Funding (source) | WO pre study | Primary outcome change | Quality* |
| --- | --- | --- | --- | --- | --- | --- | --- | --- | --- | --- | --- | --- | --- | --- | --- | --- |
| Sultan, 2024^8^ | Prospective, Observational  (3M for efficacy analyses, 6M for safety analysis) | 58 (0 at 3M, 24 at 6M; 41%) | Inhalation by vaporizer | KHIRON HK cannabis flower strain THC 20%; CBD<1% | No | 39.2 | 65.5% | PCL-C global symptom severity score | PCL-C 4 subdomains | 61.5 | 95.6% | N/A | None | N/A | -13.0† at 3M; -16.8† at 6M | 4 |
| Lynskey, 2024^9^ | Prospective, Observational  (3M) | 238 (124; 52%) | Probably inhalation of flowers- not specifically mentioned | THC-dominant flower- 74.8% at most | No | 39.6 | 71.7% | PCL-C | PHQ-9, EQ-5D-5L, General health, Sleep | 58.0 | N/A | N/A | Several medical cannabis companies (commercial) | N/A | -11.0† | 5 |
| Vaddiparti, 2023^10^ | Prospective, Observational  (70 days) | 16 (1; 6%) | Mostly (>70%) inhaled | THC-dominant flower->85.7% | No | 44.0 | 40% | PCL-5 | Nightmares (PCL-5), PSQI, PANAS, Global health-physical and mental health | 49.6 | N/A | N/A | State University System of Florida (academia) | N/A | -19.3 30-days outcome,  -20.6 70-days outcome† | 5 |
| Stack, 2023^11^ | Prospective, Observational  (Median 154.4D for efficacy analyses, 55.8D for safety analysis) | 57 in the efficacy analyses, 158 in the safety analyses (N/A; N/A%) | Oral | Various MC formulations | No | N/A | N/A | PROMIS-29 | PROMIS-29 sub-domains | N/A | N/A | N/A | Applied Cannabis Research (Private clinical research organization) | N/A | Anxiety†, depression†, fatigue†,  social abilities† | 2 |
| Pillai, 2022^12^ | Prospective, Observational  (6M) | 162 (111; 68%) | Most (49.4%) flowers vaporizing, 29.6% combined with oil extract and 11.7% only via oil extract | Mainly Rich-THC formulations (based on median doses) | No | 37.6 | 60% | N/A | IES-R, EQ-5D-5L, SQS, GAD-7, PGIC | N/A | 88.9% | Antidepressants (n=124), Benzodiazepines (n=28), Insomnia-related (n=14) | None | N/A | Significant improvement in all secondary outcome measures† | 5 |
| Sznitman, 2022^13^ | Prospective, Observational  (15D) | 92 (15; 40%) | N/A | Mainly Rich-THC formulations (based on median doses) | No | 40.4 | 55.8% | PCL-5 | Number of awakening, nightmares presence, early wakening | N/A | 100% | N/A | Evelyn Lipper Foundation (Private nonprofit foundation) | N/A | Significant† improvement in the nightmare when MC was consumed closer to the time of sleep | 3 |
| Bonn-miller, 2022^14^ | Prospective, Observational  (12M) | 150 (49; 33%) | Primarily smoked flower cannabis | THC-rich: Flowers 91% | Yes | 50.7 | 73% | CAPS-5 | PSQI, ISI, IPF, Actigraphy (SOL, SE, WASO, NWAK, TST, IPAQ) | 33.54 (Cannabis group), 35.70 (Control group) | 100% | N/A | Colorado Department of Public Health and Environment (government) | No | Cannabis users reporting a significantly† greater (-9.58) rate of decline over time compared to controls (-5.7) | 6 |
| Chan, 2017^15^ | Prospective, Observational  (10M) | 588 (N/A; N/A%) | N/A | Various MC formulations | No | 43.2 | 78% | QOLS total score | Pain intensity, GCI, QOLS sub domains | Most reported fair-bad QoL | 78% | N/A | Several Private nonprofit foundations, MedReleaf (commercial) | N/A | Significant† improvement and most reported on good-fair QoL | 3 |
| Roitman, 2014^16^ | Prospective, Observational  (3W) | 10 (N/A; N/A%) | Oral | 5 mg of D9-THC | No | 52.3 | 70% | CAPS-5 | CGI, CGI-S, CGI-I, PSQI, NFQ, NES | 94.0 | N/A | Stable:  Duloxetine 30%, Escitalopram 30%, Mirtazapine 20%, Bupropion 20%, Clonazepam 50%, Lorazepam 30% | Not reported | month not used was required | -16.0 | 4 |

CAPS-5, Clinician-Administered PTSD Scale for DSM-5; CBD, Cannabidiol; D, day; EQ-5D-5L, EuroQol 5 Dimensions 5 Levels Questionnaire; GAD-7, Generalized Anxiety Disorder 7-Item Scale; GCI, Global Clinical Impression; IES-R, Impact of Event Scale – Revised; IPAQ, International Physical Activity Questionnaire; IPF, Inventory of Psychosocial Functioning; ISI, Insomnia Severity Index; M, month; MC, medical cannabis; N/A, Not Applicable; NES, Negative Effects Scale; NFQ, Nijmegen Fatigue Questionnaire; NWAK, number of awakenings; PANAS, Positive and Negative Affect Schedule; PCL-5, PTSD Checklist for DSM-5; PCL-C, PTSD Checklist – Civilian Version; PGIC, Patient Global Impression of Change; PHQ-9, Patient Health Questionnaire-9; PSQI, Pittsburgh Sleep Quality Index; QoL, quality of life; SOL, sleep onset latency; SQS, Sleep Quality Scale; THC, Δ⁹-Tetrahydrocannabinol; TST, total sleep time; W, week; WAS, wake after sleep onset; WO, washout; *, Quality based on NOS scale, †, Significant improvement from baseline; M, Months.

**Supplementary Table 5:** **Summary of prospective observational studies safety outcomes**

| **First author, year** | **Adverse events n/N (%)** | **Adverse events n/N (%) by SOC** |
| --- | --- | --- |
| Sultan, 2024^8^ | No adverse event (AE) was reported | No adverse event (AE) was reported |
| Lynskey, 2024^9^ | Not described | Not described |
| Stack, 2023^11^ | N/A- not described for the PTSD cohort | N/A- not described for the PTSD cohort |
| Vaddiparti, 2023^10^ | Not described | Not described |
| Chan, 2017^15^ | Dry mouth 23/115 (20.0%), Feeling "high" 15/115 (13.0%), Sleepiness 14/115 (12.2.0%), Red/irritated eyes 9/115 (7.8%), Palpitations 7/115 (6.1%), Decreased memory 7/115 (6.1%). | Gastrointestinal disorders 23/115 (20.0%)  Nervous system disorders 21/115 (18.3%)  Eye disorders 9/115 (7.8%)  Cardiac disorders 7/115 (6.1%) |
| Pillai, 2022^12^ | Reported by cases and not by patients:  Abdominal pain (upper) 1/220 (0.5%), Amnesia 5/220 (2.3%), Anorexia 6/220 (2.7%), Anxiety 5/220 (2.3%), Arthritis 1/220 (0.5%), Ataxia 4/220 (1.8%), Blurred vision 4/220 (1.8%), Cognitive Disturbance 7/220 (3.2%), Confusion 7/220 (3.2%), Concentration impairment 14/220 (6.4%), Constipation 2/220 (0.9%), Delirium 3/220 (1.4%), Decreased Weight 8/220 (3.6%), Diarrhea 1/220 (0.5%), Distorted thoughts 1/220 (0.5%), Dizziness 8/220 (3.6%), Dry mouth 14/220 (6.3%), Dysgeusia 3/220 (1.4%), Dyspepsia 4/220 (1.8%), Fatigue 20/220 (9.0%), Headache 15/220 (6.8%), Insomnia 20/220 (9.0%), Intrusive thoughts 1/220 (0.5%), Irritability 2/220 (0.9%), Lethargy 13/220 (5.9%), Muscular Weakness 5/220 (2.3%), Nausea 9/220 (4.1%), Nightmares 2/220 (0.9%), Paranoia 1/220 (0.4%), Pharyngitis 4/220 (1.8%), Pyrexia 1/220 (0.5%), Rash 2/220 (0.9%), Respiratory Infection 1/220 (0.5%), Somnolence 13/220 (5.9%), Spasticity 2/220 (0.9%), Tremor 4/220 (1.8%), Vertigo 5/220 (2.3%), Vomiting 2/220 (0.9%) | Nervous system disorders: 90/220 (40.9%)  Psychiatric disorders: 40/220 (18.2%)  Gastrointestinal disorders: 33/220 (15.0%)  General disorders and administration site conditions: 26/220 (11.8%)  Investigations: 8/220 (3.6%)  Metabolism and nutrition disorders: 6/220 (2.7%)  Skin and subcutaneous tissue disorders: 2/220 (0.9%)  Musculoskeletal and connective tissue disorders: 1/220 (0.5%) |
| Sznitman, 2022^13^ | Not described |  |
| Bonn-miller, 2022^14^ | Not described |  |
| Roitman, 2014^16^ | Dry mouth 2/10 (20.0%), Headache 1/10 (10.0%), Tremor 1/10 (10.0%) | Gastrointestinal disorders 2/10 (20.0%)  Nervous system disorders 2/10 (20.0%) |

AE, Adverse event; N/A, Not Applicable; PTSD, Post-traumatic Stress-Disorder; SOC, System organ class.

**Supplementary Table 6:** **Summary of retrospective observational studies characteristics and efficacy results**

| First author, year | Design (duration of treatment) | N enrolled (N and % dropout) | Administration route | Intervention drug | Control group | Age (M, years) | Male (%) | Method of primary outcome | Method of secondary outcomes | Baseline  primary outcome | Previous cannabis experience | Background treatment | Funding (source) | WO pre study | Primary outcome change | Quality* |
| --- | --- | --- | --- | --- | --- | --- | --- | --- | --- | --- | --- | --- | --- | --- | --- | --- |
| Nacasch, 2023^17^ | Retrospective, Observational  (Mean FU of 1.1Y, range 0.5 to 3Y) | 14 (N/A; N/A%) | N/A | Not more than 20 gr of MC | No | 49.5 | 86% | PSQI | PDS | 14.6 | N/A | N/A | Not reported | N/A | -4.5† | 4 |
| Meakin, 2020^18^ | Retrospective, Observational (<6 to >24 M) | 60 (13; 27%) | Oral (capsule) | Nabilone (range of 0.5 mg to 8 mg daily) | No | N/A | N/A | A modified Clinical Global Impression Scale | N/A | N/A | N/A | N/A | None | N/A | 73% reported a marked improvement in their symptoms, with complete, or nearly complete, remission of all symptoms as rated | 2 |
| Lafrance, 2020^19^ | Retrospective case series (31M) | 404 (N/A; N/A%) | N/A | Unspecified cannabis, mostly THC-rich | No | 39.7 | 43% | Symptoms severity (0-10): Intrusions, Flashbacks,  Irritability,  Anxiety | N/A | N/A | N/A | N/A | Washington State University (academia) | N/A | Intrusive thoughts, Flashbacks, Irritability and Anxiety.  reduced in tracked sessions (> 97%), 91%, 97% and 93%, respectively | 3 |
| Elms, 2019^20^ | Retrospective case series (8W) | 21(7; 33%) 11 in the efficacy analysis | Oral - capsule or liquid spray | CBD average initial daily dose 33.18 mg | No | 39.9 | 27% | PCL-C | N/A | 51.82 | N/A | Anticonvulsant 54.5%, Antidepressant 54.5%, Antipsychotic 18.2%, Anxiolytic/sedative 54.6%, Beta-blocker 36.4% | Not reported | N/A | -14.7 | 2 |
| Smith, 2017^21^ | Retrospective case series (3-18M) | 100 (N/A; N/A%) | N/A | Various MC formulations avg of 9.4gr per day | No | 43 | 97% | PTSD symptoms severity aggregated score | PTSD symptoms subdomains, Pain intensity, Changes in other PTSD related medications | 7.0 | N/A | N/A | Several Private nonprofit foundations, MedReleaf (commercial) | N/A | -4.1† | 3 |
| Wilkinson, 2015^22^ | Retrospective, Observational  (4M) | 831 (93; 11%) "Starters" group only | N/A | Cannabis | No | 51.7 | 97.8% | SF-MISS | Violence, Employment status | 40.2 | No | Psychotropic medication 86.6% | National Institute of Mental Health (government) | N/A | -0.6 | 4 |
| Greer, 2014^23^ | Retrospective, Observational  (N/A) | 80 (N/A; N/A%) | N/A | Unspecified cannabis | No | N/A | N/A | CAPS-5 | N/A | 98.8 | N/A | N/A | Not reported | Cannabis naive | Significant† reduction in total score -75.3 | 1 |
| Cameron, 2014^24^ | Retrospective, Observational  (Mean 11.2W, Range of 1D to 36W) | 104 (20; 19%) | Oral- powder mixed with water | Nabilone (mean initial daily dose 1.4 mg up to 6.0 mg) | No | 32.7 | 100% | PCL-C | GAF | 54.7 | 91% | N/A | None | Cannabis naive | -15.9† | 4 |
| Fraser, 2009^25^ | Retrospective, Observational  (N/A) | 47 (3; 6%) | Oral | Nabilone (range of 0.2 mg to 4.0 mg nightly) | No | 44.0 | 43% | Nightmares frequency | Discontinuation of medications | N/A | N/A | one or more psychiatric medications that they were already taking for 2 years or more | Not reported | N/A | 72% experienced total cessation or lessening of severity of nightmares (28 patients had total cessation of nightmares and 6 had satisfactory reduction) | 2 |

CAPS-5, Clinician-Administered PTSD Scale for DSM-5; CBD, Cannabidiol; D, day; EQ-5D-5L, EuroQol 5 Dimensions 5 Levels Questionnaire; GAD-7, Generalized Anxiety Disorder 7-Item Scale; GCI, Global Clinical Impression; IES-R, Impact of Event Scale – Revised; IPAQ, International Physical Activity Questionnaire; IPF, Inventory of Psychosocial Functioning; ISI, Insomnia Severity Index; M, month; MC, medical cannabis; N/A, Not Applicable; NES, Negative Effects Scale; NFQ, Nijmegen Fatigue Questionnaire; NWAK, number of awakenings; PANAS, Positive and Negative Affect Schedule; PCL-5, PTSD Checklist for DSM-5; PCL-C, PTSD Checklist – Civilian Version; PGIC, Patient Global Impression of Change; PHQ-9, Patient Health Questionnaire-9; PSQI, Pittsburgh Sleep Quality Index; QoL, quality of life; SOL, sleep onset latency; SQS, Sleep Quality Scale; THC, Δ⁹-Tetrahydrocannabinol; TST, total sleep time; W, week; WAS, wake after sleep onset; WO, washout; *, Quality based on NOS scale, †, Significant improvement from baseline.

**Supplementary Table 7:** **Summary of retrospective observational studies safety outcomes**

| **First author, year** | **Adverse events n/N (%)** | **Adverse events n/N (%) by SOC** |
| --- | --- | --- |
| Nacasch, 2023^17^ | Not described |  |
| Meakin, 2020^18^ | Reported by severity and not by patients:  Mild 24/52 (46.1%), Moderate 11/52 (21.1%), Important and interfering with function 4/52 (7.9%) |  |
| ^19^ | Not described |  |
| Elms, 2019^20^ | Fatigue 2/21 (9.5%), daytime fogginess 1/21 (4.8%), gastrointestinal pain 2/21 (9.5%), impaired concentration the 1/21 (4.8%), reflux worsening 1/21 (4.8%), | Gastrointestinal disorders: 3/21 (14.3%)  Nervous system disorders: 2/21 (9.5%)  General disorders and administration site conditions: 2/21 (9.5%) |
| Smith, 2017^21^ | Not described |  |
| Wilkinson, 2015^22^ | Not described |  |
| Greer, 2014^23^ | Not described |  |
| Cameron, 2014^24^ | Psychosis 2/104 (1.9%), Sedation 13/104 (12.5%), Dry mouth 7/104 (6.7%), Feeling "stoned" 4/104 (3.8%/), Orthostatic hypotension 2/104 (1.9%), Agitation 2/104 (1.9%), and headache 1/104 (1.0%) | Nervous system disorders: 14/104 (13.5%)  Psychiatric disorders: 4/104 (3.8%), Gastrointestinal disorders: 7/104 (6.7%)  Vascular disorders: 2/104 (1.9%) |
| Fraser, 2009^25^ | 28% reported any AE, with undescribed frequency included:  Lightheadedness, forgetfulness, dizziness, and headache |  |

AE, Adverse Event; SOC, System organ class.

**Supplementary Table 8: Jadad quality scores for RCTs**

| **First Author and Year (Clinical trial registration number)** | **Randomization (1 point) + Method (1 point)** | **Blinding (1 point) + Method (1 point)** | **Withdrawals and Dropouts (1 point)** | **Total Score (0-5)** |
| --- | --- | --- | --- | --- |
| Zabik, 2024 ^1^ (N/A) | 2 | 1 | 1 | 4 |
| Zabik, 2023^2^ (N/A) | 2 | 1 | 1 | 4 |
| Walsh, 2023^3^ (NCT02517424) | 1 | 1 | 1 | 3 |
| Bolsoni, 2022^4^ (N/A) | 2 | 2 | 0 | 4 |
| Bolsoni, 2022^5^ (N/A) | 2 | 2 | 0 | 4 |
| Bonn-Miller, 2021 ^6^ (N/A) | 2 | 1 | 1 | 4 |
| Jetly, 2015^7^ (N/A) | 1 | 2 | 1 | 4 |

**Supplementary Table 9: Newcastle–Ottawa Scale for observational studies evaluation**

| **Author name and year of publication** | **Research Question Clearly Stated** | **Study Population Defined** | **Uniform Selection/Recruitment** | **Sample Size Justification** | **Exposure Measured Before Outcome** | **Sufficient Timeframe** | **Levels of Exposure Examined** | **Exposure Measures Valid/Reliable** | **Repeated Exposure Assessment** | **Outcome Measures Valid/Reliable** | **Outcome Assessors Blinded** | **Follow-Up ≤20% Loss** | **Confounders Measured/Adjusted** | **Total score** |
| --- | --- | --- | --- | --- | --- | --- | --- | --- | --- | --- | --- | --- | --- | --- |
| **Prospective observational studies** | | | | | | | | | | | | | | |
| Sultan, 2024^8^ | Yes | Yes | Yes | No | NR | Yes | No | No | Yes | Yes | No | No | NR | 3 |
| Lynskey, 2024^9^ | Yes | Yes | Yes | No | NR | Yes | No | NA | No | Yes | NR | No | Yes | 5 |
| Vaddiparti, 2023^10^ | Yes | Yes | Yes | No | Yes | No | Yes | No | Yes | Yes | NR | Yes | No | 5 |
| Stack, 2023^11^ | Yes | Yes | CD | No | Yes | CD | No | NA | No | Yes | NR | NR | No | 3 |
| Pillai, 2022^12^ | Yes | Yes | Yes | No | Yes | Yes | No | NA | Yes | Yes | NR | No | No | 5 |
| Sznitman, 2022^13^ | Yes | Yes | Yes | No | Yes | No | No | No | Yes | Yes | No | Yes | Yes | 4 |
| Bonn-miller, 2022^14^ | Yes | Yes | Yes | Yes | Yes | Yes | Yes | No | Yes | Yes | NR | No | Yes | 7 |
| Chan, 2017^15^ | Yes | No | No | No | Yes | Yes | No | NA | Yes | CD | NR | No | No | 3 |
| Roitman, 2014^16^ | Yes | Yes | Yes | No | Yes | No | No | Yes | Yes | Yes | NR | Yes | NR | 4 |
| **Retrospective observational studies** | | | | | | | | | | | | | | |
| Nacasch, 2023^17^ | No | Yes | Yes | No | Yes | Yes | No | No | No | Yes | CD | Yes | No | 4 |
| Meakin, 2020^18^ | Yes | Yes | Yes | NR | No | Yes | No | Yes | No | CD | CD | No | No | 3 |
| Lafrance, 2020^19^ | Yes | Yes | CD | NR | CD | Yes | No | CD | Yes | No | CD | CD | Yes | 3 |
| Elms, 2019^20^ | Yes | Yes | CD | NR | No | No | Yes | Yes | Yes | Yes | CD | CD | No | 3 |
| Smith, 2017^21^ | Yes | Yes | Yes | No | Yes | Yes | Yes | Yes | No | No | No | CD | No | 3 |
| Wilkinson, 2015^22^ | Yes | Yes | Yes | No | Yes | Yes | No | No | CD | Yes | No | Yes | Yes | 5 |
| Greer, 2014^23^ | Yes | Yes | No | No | CD | CD | NR | No | CD | Yes | CD | NA | No | 2 |
| Cameron, 2014^24^ | Yes | Yes | Yes | Yes | Yes | CD | Yes | Yes | No | Yes | CD | CD | No | 4 |
| Fraser, 2009^25^ | No | Yes | Yes | No | CD | Yes | No | Yes | CD | No | No | No | No | 2 |

Possible answer: (Yes/No, CD = Cannot Determine; NR = Not Reported; NA = Not Applicable); *Possible total score: low quality (0-3), moderate quality (4-6), high quality (7-9)

**Supplemental Table 10: Summary of Studies Identified from ClinicalTrials.gov without published Results**

| **Principal investigator/Responsible Party (NCT Number) and estimated completion** | **Study Title** | **Study Status** | **Interventions** | **Study Design** | **Duration** | **Primary outcome** |
| --- | --- | --- | --- | --- | --- | --- |
| Arieh Y. Shalev (NCT00965809) 2013-04 | Add on Study on delta-9-THC Treatment for Posttraumatic Stress Disorders (PTSD) | Unknown | 1. THC 5 mg in olive oil. 2. Placebo in a matched vial- only olive oil | Randomized, parallel, placebo controlled, Triple blinded | 6W | CAPS-5 |
| Margaret Haney  (NCT03251326) 2019-06 | Nabilone in Cannabis Users With PTSD | Terminated | 1.Nabilone  capsules (4 mg)  2. Propranolol  capsules (40mg)  3.Smoked cannabis  (0.0 and 5.6% THC)  4. Placebo capsules | Randomized, crossover, placebo controlled, Double blinded | 1M | N/A |
| Christine Rabinak  (NCT04080427) 2025-12-31 | Effects of Delta9-tetrahydrocannabinol (THC) on Retention of Memory for Fear Extinction Learning in PTSD: R33 Study | Recruiting | 1. Dronabinol 7.5 milligram oral capsule 2. Placebo capsule | Randomized, parallel, placebo controlled, double blinded | 3M | fMRI, SCR, PCL-5, CAPS-5, SUDS |
| Michael J. Telch  (NCT04197102) 2025-05 | Use of CBD Oil in the Treatment of Posttraumatic Stress Disorder | Suspended | 1. 300 mg/day of CBD isolate. 2. 300 mg/day of CBD Broad Spectrum Oil  3. Matched Placebo Oil | Randomized, parallel, placebo controlled, Quadruple blinded | 13W | PCL-5 |
| Stefan Roepke  (NCT04448808) 2025-05 | Treating Nightmares in Posttraumatic Stress Disorder with Dronabinol | Recruiting | 1. BX-1 (dronabinol)  2. Placebo | Randomized, parallel, placebo controlled, Quadruple blinded | 10W | Frequency and intensity of nightmares, measured with the CAPS-5 B2 score |
| Esther M Blessing, Charles R Marmar  (NCT04550377) 2026-06 | Cannabidiol as a Treatment for PTSD and PTSD Comorbid With TBI | Recruiting | 1) Oral CBD 400 mg daily  2) Oral CBD 800 mg daily  3) Placebo daily | Randomized, parallel, placebo controlled, double blinded | 8W | CAPS-5 |
| Jazz Pharmaceuticals  (NCT04592159) 2023-03 | Study of the Safety and Efficacy of Nabiximols Oromucosal Spray Versus Placebo in Patients with Post-traumatic Stress-Disorder | Withdrawn | 1. Nabiximols Each spray delivers 100 microliters (μL) of nabiximols. Max 12 sprays per day  2. Placebo oromucosal spray | Randomized, parallel, placebo controlled, triple blinded | 8W | CAPS-5 |
| Matthew Rizzo  (NCT05269459) 2029-04 | Safety and Efficacy of Cannabidiol (CBD) for Symptoms of PTSD in Adults | Recruiting | 1. CBD as Nantheia ATL5 400 mg twice a day 2. Placebo Matching gel capsules containing no active drug | Randomized, parallel, placebo controlled, double blinded | 8W | CAPS-5 |
| Leslie Lundahl  (NCT06381180) 2030-12-31 | Warrior CARE: Cannabis Behavioral Health (CBH) | Not yet recruiting | 1. THC-rich plant cannabis that will be vaporized: Cannabis with 2.5 mg THC; up to a maximum of 5 doses/day (max dose = 12.5 mg THC). 2. CBD-rich plant that will be vaporized: Cannabis with 2.5 mg CBD; up to a maximum of 5 doses/day (max dose = 12.5 mg CBD)  3. THC & CBD equal cannabis plant with 2.5 mg THC and 2.5 mg CBD; up to a maximum of 5 doses/day (max doses = 12.5 mg THC and 12.5 mg CBD). 4. Placebo cannabis with <1mg THC and <1mg CBD; up to a maximum of 5 doses/day (max dose = <5 mg THC and <5 mg CBD). | Randomized, parallel, placebo controlled, double blinded | 12W | CAPS-5,  PCL-5,  C-SSRS,  SBQ-R,  BDI-II, STAI-Y |

BDI-II, Beck Depression Inventory-II; CAPS-5, Clinician-Administered PTSD Scale for DSM-5; CBD, cannabidiol; fMRI, functional magnetic resonance imaging; N/A, not applicable; NCT, National Clinical Trial number; PCL-5, PTSD Checklist for DSM-5; SBQ-R, Suicide Behaviors Questionnaire-Revised; SCR, Skin Conductance Response; SSRS, Social Support Rating Scale; SUDS, Subjective Units of Distress Scale; STAI-Y, State-Trait Anxiety Inventory-Y; THC, tetrahydrocannabinol; W, weeks.

**Supplemental Table 11: Summary of Studies Identified from ClinicalTrials.gov with Peer-reviewed Published Results**

| **Principal investigator/Responsible Party (NCT Number) and estimated completion** | **Study Title** | **Study Status** | **Interventions** | **Study Design** | **Duration** | **Primary outcome** |
| --- | --- | --- | --- | --- | --- | --- |
| Christine A. Rabinak  (NCT02069366) 2019-12 estimated completion | Cannabinoid Control of Fear Extinction Neural Circuits in Post-traumatic Stress Disorder | Completed | 1. Dronabinol 7.5 mg in olive oil. 2. Placebo in a matched vial- only olive oil | Randomized, parallel, placebo controlled, Double blinded | 4 consequent visits during 15 days | fMRI measure |
| Zach Walsh  (NCT02517424) 2019-03-22 | Evaluating Safety and Efficacy of Cannabis in Participants With Chronic Posttraumatic Stress Disorder | Completed | 1. Vaporized High THC/Low CBD Cannabis up to 2 gr/day  2.Vaporized High THC/High CBD Cannabis up to 2 gr/day  3. Vaporized Low THC/Low CBD Cannabis up to 2 gr/day | Randomized, crossover, placebo controlled, Triple blinded | 3 weeks | CAPS-5 |
| Sue Sisley  (NCT02759185) 2019-01 | Pilot Study of the Safety and Efficacy of Four Different Potencies of Smoked Marijuana in 76 Veterans With PTSD | Completed | 1. High THC cannabis up to 1.8 gr/day  2. High CBD cannabis up to 1.8 gr/day  3. THC/CBD cannabis up to 1.8 gr/day  4. Placebo cannabis | Randomized, crossover, placebo controlled, Triple blinded | 6 weeks | CAPS-5 |
| Christine A. Rabinak  (NCT03008005) 2019-12-31 | Effects of Delta-9 Tetrahydrocannabinol (THC) on Retention of Memory for Fear Extinction Learning in PTSD: R61 Study | Completed | 1. Placebo Oral Capsule  2. Drug: Dronabinol Cap 5 milligrams (MG)  3. Drug: Dronabinol Cap 10 milligrams (MG) | Randomized, parallel, placebo controlled, Double blinded | 5 consequent visits during 21 days | fMRI measure |

CAPS-5, Clinician-Administered PTSD Scale for DSM-5; CBD, cannabidiol; fMRI, functional magnetic resonance imaging; NCT, National Clinical Trial number; THC, tetrahydrocannabinol.

| **Section and Topic** | **Item #** | **Checklist item** | **Location where item is reported** |
| --- | --- | --- | --- |
| **TITLE** | | | page |
| Title | 1 | Identify the report as a systematic review. | 1 |
| **ABSTRACT** | | |  |
| Abstract | 2 | See the PRISMA 2020 for Abstracts checklist. | 2 |
| **INTRODUCTION** | | |  |
| Rationale | 3 | Describe the rationale for the review in the context of existing knowledge. | 6-8 |
| Objectives | 4 | Provide an explicit statement of the objective(s) or question(s) the review addresses. | 8 |
| **METHODS** | | |  |
| Eligibility criteria | 5 | Specify the inclusion and exclusion criteria for the review and how studies were grouped for the syntheses. | 9 |
| Information sources | 6 | Specify all databases, registers, websites, organisations, reference lists and other sources searched or consulted to identify studies. Specify the date when each source was last searched or consulted. | 9 |
| Search strategy | 7 | Present the full search strategies for all databases, registers and websites, including any filters and limits used. | 9 |
| Selection process | 8 | Specify the methods used to decide whether a study met the inclusion criteria of the review, including how many reviewers screened each record and each report retrieved, whether they worked independently, and if applicable, details of automation tools used in the process. | 10 |
| Data collection process | 9 | Specify the methods used to collect data from reports, including how many reviewers collected data from each report, whether they worked independently, any processes for obtaining or confirming data from study investigators, and if applicable, details of automation tools used in the process. | 10 |
| Data items | 10a | List and define all outcomes for which data were sought. Specify whether all results that were compatible with each outcome domain in each study were sought (e.g. for all measures, time points, analyses), and if not, the methods used to decide which results to collect. | 11 |
|  | 10b | List and define all other variables for which data were sought (e.g. participant and intervention characteristics, funding sources). Describe any assumptions made about any missing or unclear information. | 10 |
| Study risk of bias assessment | 11 | Specify the methods used to assess risk of bias in the included studies, including details of the tool(s) used, how many reviewers assessed each study and whether they worked independently, and if applicable, details of automation tools used in the process. | 10 |
| Effect measures | 12 | Specify for each outcome the effect measure(s) (e.g. risk ratio, mean difference) used in the synthesis or presentation of results. | NA |
| Synthesis methods | 13a | Describe the processes used to decide which studies were eligible for each synthesis (e.g. tabulating the study intervention characteristics and comparing against the planned groups for each synthesis (item #5)). | 10 |
|  | 13b | Describe any methods required to prepare the data for presentation or synthesis, such as handling of missing summary statistics, or data conversions. | NA |
|  | 13c | Describe any methods used to tabulate or visually display results of individual studies and syntheses. | NA |
|  | 13d | Describe any methods used to synthesize results and provide a rationale for the choice(s). If meta-analysis was performed, describe the model(s), method(s) to identify the presence and extent of statistical heterogeneity, and software package(s) used. | 10 |
|  | 13e | Describe any methods used to explore possible causes of heterogeneity among study results (e.g. subgroup analysis, meta-regression). | NA |
|  | 13f | Describe any sensitivity analyses conducted to assess robustness of the synthesized results. | NA |
| Reporting bias assessment | 14 | Describe any methods used to assess risk of bias due to missing results in a synthesis (arising from reporting biases). | NA |
| Certainty assessment | 15 | Describe any methods used to assess certainty (or confidence) in the body of evidence for an outcome. | 10 |
| **RESULTS** | | |  |
| Study selection | 16a | Describe the results of the search and selection process, from the number of records identified in the search to the number of studies included in the review, ideally using a flow diagram. | 12 |
|  | 16b | Cite studies that might appear to meet the inclusion criteria, but which were excluded, and explain why they were excluded. | 12 |
| Study characteristics | 17 | Cite each included study and present its characteristics. | Supplementary materials |
| Risk of bias in studies | 18 | Present assessments of risk of bias for each included study. | Supplementary materials |
| Results of individual studies | 19 | For all outcomes, present, for each study: (a) summary statistics for each group (where appropriate) and (b) an effect estimate and its precision (e.g. confidence/credible interval), ideally using structured tables or plots. | Supplementary materials |
| Results of syntheses | 20a | For each synthesis, briefly summarise the characteristics and risk of bias among contributing studies. | Supplementary materials |
|  | 20b | Present results of all statistical syntheses conducted. If meta-analysis was done, present for each the summary estimate and its precision (e.g. confidence/credible interval) and measures of statistical heterogeneity. If comparing groups, describe the direction of the effect. | NA |
|  | 20c | Present results of all investigations of possible causes of heterogeneity among study results. | 12-17 |
|  | 20d | Present results of all sensitivity analyses conducted to assess the robustness of the synthesized results. | NA |
| Reporting biases | 21 | Present assessments of risk of bias due to missing results (arising from reporting biases) for each synthesis assessed. | NA |
| Certainty of evidence | 22 | Present assessments of certainty (or confidence) in the body of evidence for each outcome assessed. | Supplementary materials |
| **DISCUSSION** | | |  |
| Discussion | 23a | Provide a general interpretation of the results in the context of other evidence. | 18-20 |
|  | 23b | Discuss any limitations of the evidence included in the review. | 20 |
|  | 23c | Discuss any limitations of the review processes used. | 20 |
|  | 23d | Discuss implications of the results for practice, policy, and future research. | 18-20 |
| **OTHER INFORMATION** | | |  |
| Registration and protocol | 24a | Provide registration information for the review, including register name and registration number, or state that the review was not registered. | NA |
|  | 24b | Indicate where the review protocol can be accessed, or state that a protocol was not prepared. | NA |
|  | 24c | Describe and explain any amendments to information provided at registration or in the protocol. | NA |
| Support | 25 | Describe sources of financial or non-financial support for the review, and the role of the funders or sponsors in the review. | 22 |
| Competing interests | 26 | Declare any competing interests of review authors. | 22 |
| Availability of data, code and other materials | 27 | Report which of the following are publicly available and where they can be found: template data collection forms; data extracted from included studies; data used for all analyses; analytic code; any other materials used in the review. | 22 |

**References**

1. Zabik, N. L. *et al.* Dose-dependent effect of acute THC on extinction memory recall and fear renewal: a randomized, double-blind, placebo-controlled study. *Psychopharmacology (Berl).* 1–16 (2024). doi:10.1007/s00213-024-06702-w ORIGINAL INVESTIGATION Dose-dependent

2. Zabik, N. L., Rabinak, C. A., Peters, C. A. & Iadipaolo, A. Cannabinoid modulation of corticolimbic activation during extinction learning and fear renewal in adults with posttraumatic stress disorder. *Neurobiol. Learn. Mem.* **201**, 107758 (2023).

3. Walsh, Z. *et al.* A small clinical trial of vaporized cannabis for PTSD: suggestive results and directions for future study. *Trials* **24**, 23–26 (2023).

4. Bolsoni, L. M., Crippa, J. A. S., Hallak, J. E. C., Guimaraes, F. S. & Zuardi, A. W. The anxiolytic effect of cannabidiol depends on the nature of the trauma when patients with post-traumatic stress disorder recall their trigger event. *Brazilian J. Psychiatry* **44**, 298–307 (2022).

5. Bolsoni, L. M., Crippa, J. A. S., Hallak, J. E. C., Guimarães, F. S. & Zuardi, A. W. Effects of cannabidiol on symptoms induced by the recall of traumatic events in patients with posttraumatic stress disorder. *Psychopharmacology (Berl).* **239**, 1499–1507 (2022).

6. Bonn-Miller, M. O. *et al.* The short-term impact of 3 smoked cannabis preparations versus placebo on PTSD symptoms: A randomized cross-over clinical trial. *PLoS One* **16**, 1–26 (2021).

7. Jetly, R., Heber, A., Fraser, G. & Boisvert, D. The efficacy of nabilone, a synthetic cannabinoid, in the treatment of PTSD-associated nightmares: a preliminary randomized, double-blind, placebo-controlled cross-over design study. *Psychoneuroendocrinology* **51**, 585–588 (2015).

8. Sultan, W. & Madiedo, A. Controlled inhalation of THC-predominant cannabis flos mitigates severity of PTSD symptoms and improves quality of sleep and general mood in cannabis-experienced UK civilians: a real-world, observational study. *Med Cannabis Cannabinoids* 1–19 (2024). doi:10.1159/000540978

9. Lynskey, M. T., Athanasiou-fragkouli, A., Thurgur, H., Schlag, A. K. & Nutt, D. J. Medicinal cannabis for treating post-traumatic stress disorder and comorbid depression: real-world evidence. *BJPsych Open* **10**, 1–7 (2024).

10. Vaddiparti, K. *et al.* Improved Post-Traumatic Stress Disorder Symptoms and Related Sleep Disturbances after Initiation of Medical Marijuana Use: Evidence from a Prospective Single Arm Pilot Study. *Med. Cannabis Cannabinoids* **6**, 160–169 (2023).

11. Stack, S. K. *et al.* The Effectiveness and Adverse Events of Cannabidiol and Tetrahydrocannabinol Used in the Treatment of Anxiety Disorders in a PTSD Subpopulation: An Interim Analysis of an Observational Study. *J. Pharm. Technol.* **39**, 172–182 (2023).

12. Pillai, M. *et al.* Assessment of clinical outcomes in patients with post-traumatic stress disorder: analysis from the UK Medical Cannabis Registry. *Expert Rev. Neurother.* **22**, 1009–1018 (2022).

13. Sznitman, S. R., Meiri, D., Amit, B. H., Rosenberg, D. & Greene, T. Posttraumatic stress disorder, sleep and medical cannabis treatment: A daily diary study. *J. Anxiety Disord.* **92**, 1–7 (2022).

14. Bonn-miller, M. O. *et al.* The Long-Term, Prospective, Therapeutic Impact of Cannabis on Post-Traumatic Stress Disorder. *Cannabis Cannabinoid Res.* **7**, 214–223 (2022).

15. Chan, S. *et al.* Medical cannabis use for patients with post-traumatic stress disorder (PTSD). *J. Pain Manag.* **10**, 385–396 (2017).

16. Roitman, P., Mechoulam, R. & Shalev, R. C. A. Preliminary, Open-Label, Pilot Study of Add-On Oral Δ9-Tetrahydrocannabinol in Chronic Post-Traumatic Stress Disorder. *Clin Drug Investig* **34**, 587–591 (2014).

17. Nacasch, N., Avni, C. & Toren, P. Medical cannabis for treatment-resistant combat PTSD. *Front. Psychiatry* **13**, 1–7 (2023).

18. Meakin, C., Fraser, G., Boisvert, D. & Miller, C. Use of a synthetic cannabinoid (nabilone) in the ongoing management of posttraumatic stress disorder nightmares in the Canadian Armed Forces: Results of an anonymous online survey. *J. Mil. Veteran Fam. Heal.* **6**, 3–8 (2020).

19. Lafrance, E. M., Glodosky, N. C., Bonn-miller, M. & Cuttler, C. Short and Long-Term Effects of Cannabis on Symptoms of Post-Traumatic Stress Disorder. *J. Affect. Disord.* **274**, 298–304 (2020).

20. Elms, L., Shannon, S., Hughes, S. & Lewis, N. Cannabidiol in the Treatment of Post-Traumatic Stress Disorder: A Case Series. *J. Altern. Complement. Med.* **25**, 392–397 (2019).

21. Smith, P. A. *et al.* Medical cannabis use in military and police veterans diagnosed with post-traumatic stress disorder (PTSD). *J. Pain Manag.* **10**, 397–405 (2017).

22. Wilkinson, S. T., Stefanovics, E. & Rosenheck, R. A. Marijuana use is associated with worse outcomes in symptom severity and violent behavior in patients with posttraumatic stress disorder. *J Clin Psychiatry* **76**, 1174–1180 (2015).

23. Greer, G. R., Grob, C. S. & Halberstadt, A. L. PTSD symptom reports of patients evaluated for the New Mexico Medical Cannabis Program. *J. Psychoactive Drugs* **46**, 73–77 (2014).

24. Cameron, C., Watson, D. & Robinson, J. Use of a synthetic cannabinoid in a correctional population for posttraumatic stress disorder-related insomnia and nightmares, chronic pain, harm reduction, and other indications: A retrospective evaluation. *J. Clin. Psychopharmacol.* **34**, 559–564 (2014).

25. Fraser, G. A. The use of a synthetic cannabinoid in the management of treatment-resistant nightmares in posttraumatic stress disorder (PTSD). *CNS Neurosci. Ther.* **15**, 84–88 (2009).
